# The crystal structures of 6′-(4-chloro­phen­yl)- and 6′-(4-meth­oxy­phen­yl)-6a′-nitro-6a′,6b′,7′,9′,10′,12a′-hexa­hydro-2*H*,6′*H*,8′*H*-spiro­[ace­naphthyl­ene-1,12′-chromeno[3,4-*a*]indolizin]-2-one

**DOI:** 10.1107/S2056989019000422

**Published:** 2019-01-15

**Authors:** S. Syed Abuthahir, M. NizamMohideen, V. Viswanathan, D. Velmurugan, J. Nagasivarao

**Affiliations:** a PG & Research Department of Physics, The New College (Autonomous), Chennai 600 014, Tamil Nadu, India; b Department of Biophysics, All India Institute of medical Science, New Delhi 110 029, India; c CAS in Crystallography and Biophysics, University of Madras, Chennai 600 025, India; d GVK Biosciences Pvt. Ltd, Hyderabad 500 076, India

**Keywords:** crystal structure, spiro compounds, cyclo­addition, piperidine, nitro­gen-containing heterocycle, pyran, pyrrolidine, hydrogen bonding

## Abstract

In the title compounds, C_32_H_25_ClN_2_O_4_ (I) and C_33_H_28_N_2_O_5_ (II), the six-membered pyran and piperidine rings adopt envelope and chair conformations, respectively. The five-membered pyrrolidine rings adopt twist conformations. Compound (II) crystallizes with two independent mol­ecules (*A* and *B*) in the asymmetric unit. In all three mol­ecules there is a C—H⋯O intra­molecular hydrogen bond present enclosing an *S*(7) ring motif. In (I), both oxygen atoms of the nitro group are disordered, while in (II) the meth­oxy­benzene group is disordered in mol­ecule *B*. The geometries were regularized by soft restraints. In the crystal of (I), mol­ecules are linked by C—H⋯O hydrogen bonds, forming chains along [010]. The chains are linked by C—H⋯Cl hydrogen bonds, forming layers parallel to (10). Within the layer there are C—H⋯π inter­actions present. In the crystal of (II), the *A* and *B* mol­ecules are linked *via* C—H⋯O hydrogen bonds, forming a square four-membered *A*–*B*–*A*–*B* unit. These units are linked by a number of C—H⋯π inter­actions, forming a three-dimensional supra­molecular structure.

## Abstract 2

The conformations of the title compounds, (I) and (II), are very similar. The pyran rings adopt envelope conformations, the piperidine rings have chair conformations and the pyrrolidine rings adopt twist conformations. Intra- and inter­molecular C—H⋯O hydrogen bonds occur. Compound (II) crystallizes with two independent mol­ecules in the asymmetric unit which are linked by C—H⋯O hydrogen bonds.

## Chemical context   

Nitro­gen-containing heterocyclic compounds are reported to possess a diverse range of biological activities such as anti­microbial, anti­tumor and anti-inflammatory (Thirunavukkarsu *et al.*, 2017 ▸) properties. Spiro compounds are encountered in many pharmacologically active alkaloids (NizamMohideen *et al.*, 2009*c* ▸; Cravotto *et al.*, 2001 ▸). The cornerstone for cyclo­addition reactions, nitro­nes, are excellent spin-trapping and highly versatile synthetic inter­mediates (Bernotas *et al.*, 1996 ▸; NizamMohideen *et al.*, 2009*b* ▸). Highly substituted spiro compounds result from the 1,3-dipolar cyclo­addition of exocylic olefins with nitro­nes and these spiro compounds have also been transformed into complex heterocycles (Hossain *et al.*, 1993 ▸; NizamMohideen *et al.*, 2009*a* ▸). Recognizing the importance of such compounds in drug discovery and our ongoing research on the construction of novel heterocycles has prompted us to investigate the title compounds and we report herein on their synthesis and crystal structures.

## Structural commentary   

**Figure 1:**
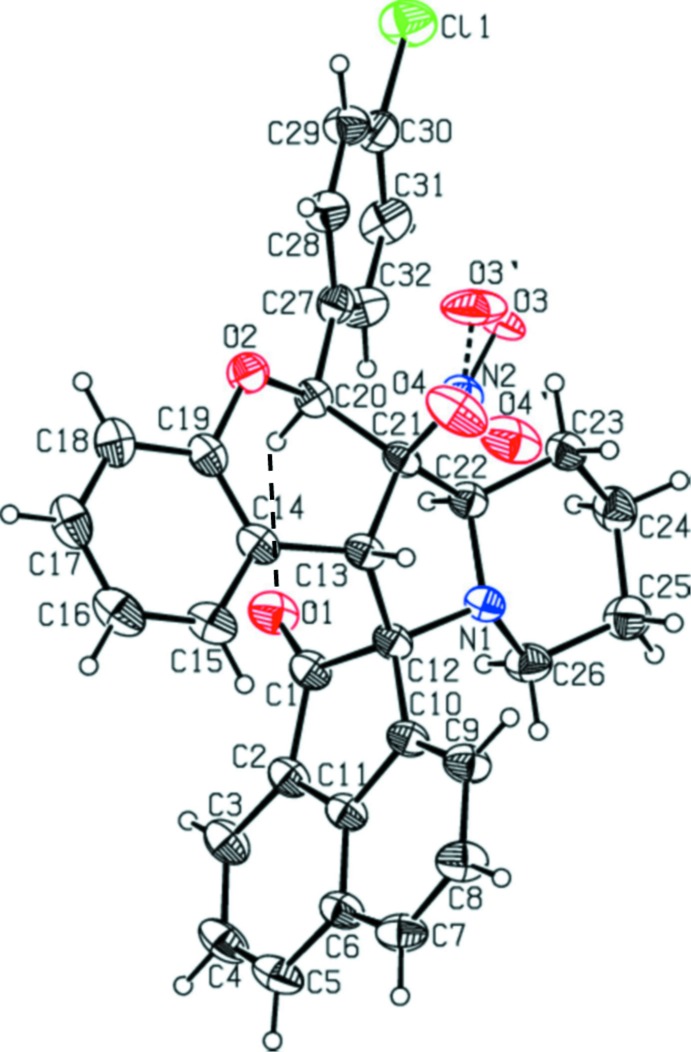
View of the mol­ecular structure of compound (I), with the atom labelling. Displacement ellipsoids are drawn at the 50% probability level. The intra­molecular C—H⋯O hydrogen bond (see Table 1 ▸) is shown as a dashed line.

**Figure 2:**
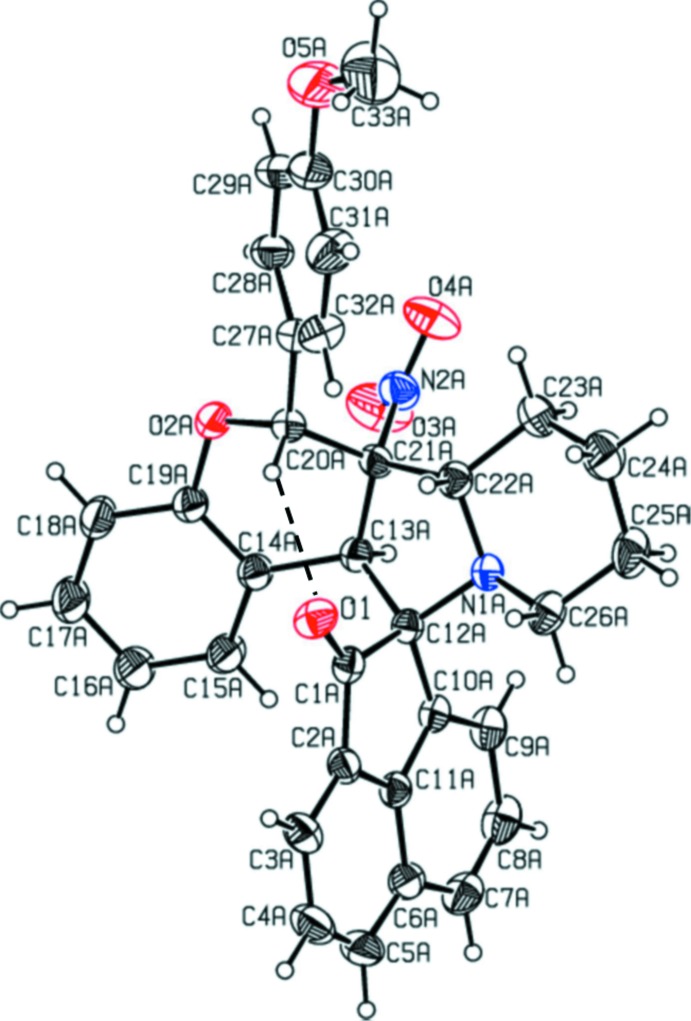
View of the mol­ecular structure of mol­ecule *A* of compound (II), with the atom labelling. Displacement ellipsoids are drawn at the 30% probability level. The intra­molecular C—H⋯O hydrogen bond (see Table 2 ▸) is shown as a dashed line.

**Figure 4:**
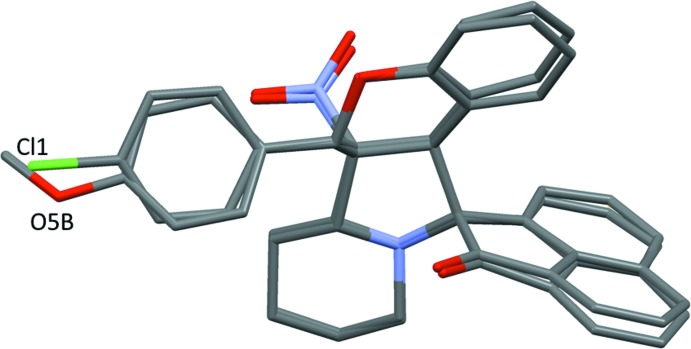
The structural overlay of compound (I) on the major component of mol­ecule *B* of compound (II).

**Figure 5:**
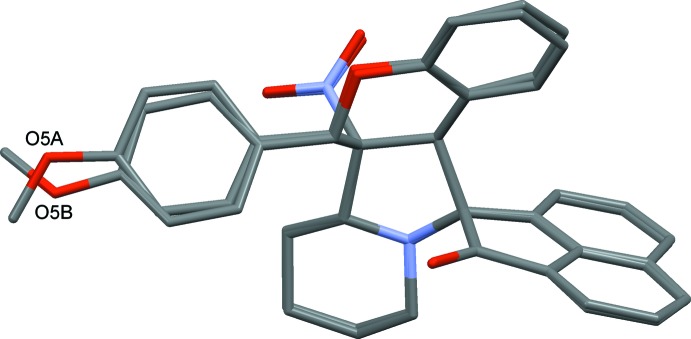
The mol­ecular overlay of inverted mol­ecule *B* (major component) on mol­ecule *A* of compound (II).

The mol­ecular structure of compound (I) is shown in Fig. 1 ▸, while the mol­ecular structures of the two independent mol­ecules, *A* and *B*, of compound (II) are shown in Figs. 2 ▸ and 3 ▸, respectively; they are in fact enanti­omers. The bond lengths and angles in all three mol­ecules are very similar. In (II), the meth­oxy­benzene group of mol­ecule *B* is positionally disordered and only the major component will be taken into consideration concerning the conformation of the mol­ecule. The structural overlap of compound (I) on the major component of mol­ecule *B* of compound (II) is shown in Fig. 4 ▸. The two mol­ecules have an r.m.s. deviation of 0.212 Å. The mol­ecular overlap of inverted mol­ecule *B* of compound II (major component) on mol­ecule *A* is shown in Fig. 5 ▸. Here the r.m.s. deviation is 0.297 Å and it can be seen that the major difference between the two mol­ecules concerns the orientation of the 4-meth­oxy­phenyl group. In all three mol­ecules (I and II*A* and II*B*) the pyran rings have envelope conformations with the methyl­ene C atom C21 as the flap. The piperidine rings adopt chair conformations, while the pyrrolidine rings adopt twist conformations on the N1—C12 bond (N1*A*—C12*A* in II*A* and N1*B*—C12*B* in II*B*).
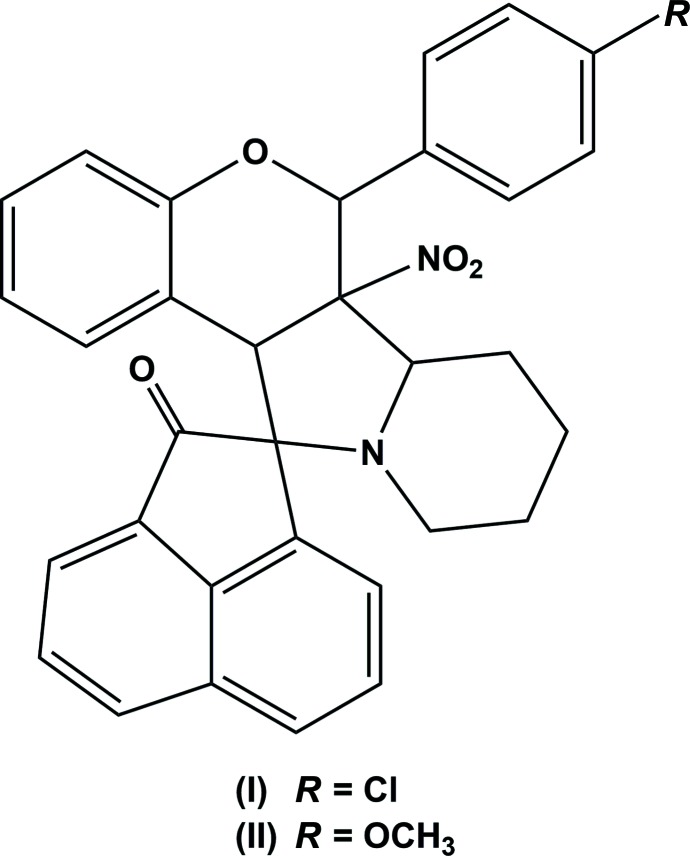


The mean plane of the five-membered pyrrolidine ring (N1/C12/C13/C21/C22) is inclined to the mean plane of the cyclo­pen3–en-1-one ring (C1/C2/C10–C12) by 85.7 (1)° in compound (I), and the equivalent dihedral angles in mol­ecules *A* and *B* of compound (II) are 87.5 (1) and 89.3 (1)°, respectively. In compound (I) the dihedral angles between the ace­naphthyl­ene ring system (C1–C12) and the benzene rings (C14–C19 and C27–C32) are 73.1 (1) and 57.3 (1)°, respectively. In (II) the corresponding dihedral angles are, respectively, 65.1 (2) and 53.6 (2)° for mol­ecule *A* and 66.7 (2) and 59.3 (5)° for mol­ecule *B*. The benzene rings (C27–C32 and C14—C19) are inclined to each other by 50.0 (1)° in (I), and 62.2 (2)° in mol­ecule *A* and 71.6 (2)° in mol­ecule *B* of (II). The mean plane of the pyrrolidine ring (N1/C12/C13/C21/C22) makes a dihedral angle with the mean plane of the pyran ring (O2/C13/C14/C219–C21) of 30.2 (2)° in (I), and 33.2 (2) for mol­ecule *A* and 36.1 (2)° for mol­ecule *B* in (II), and is inclined to the piperidine ring mean plane (N1/C22–C26) by 9.9 (2)° in (I), and 11.1 (2)° in mol­ecule *A* and 13.1 (2)° in mol­ecule *B* of compound (II). The mean planes of the pyran and piperidine ring are inclined to each other by 29.1 (2)° in (I), and 33.5 (2) in mol­ecule *A* and 36.2 (2)° in mol­ecule *B* of compound (II). Full details of the puckering parameters and lowest displacement asymmetry parameters are given in the supporting information. The keto atom O1 deviates from the mean plane of the plane of the ace­naphthyl­ene ring system (C1—C12) by 0.070 (2) Å in (I), and by 0.049 (2) and 0.162 (1) Å, respectively, in mol­ecules A and B of compound (II). Chlorine atom Cl1 deviates by 0.109 (2) Å from the plane of the benzene ring (C27–C32) in (I). It can be seen that the conformations and the values of the dihedral angles in all three mol­ecules of the title compounds are very similar. The bond lengths and angles are also close to those reported for similar compounds (Devi *et al.*, 2013*a* ▸,*b* ▸).

## Supra­molecular features   

**Figure 6:**
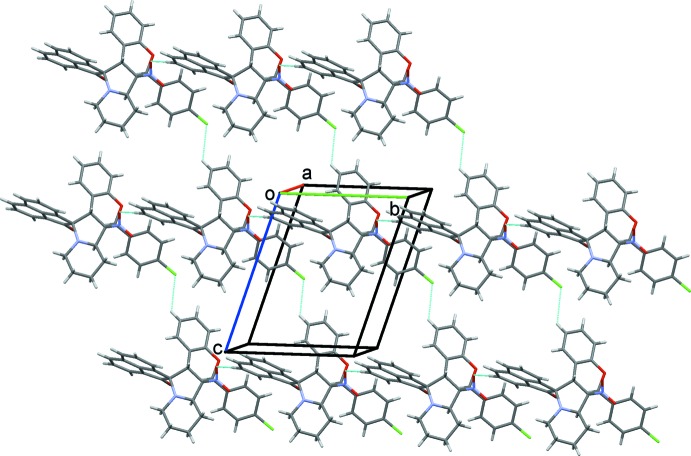
A view normal to plane (10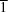) of the crystal structure of (I), showing the C—H⋯O and C—H⋯Cl hydrogen bonds (dashed lines; see Table 1 ▸).

For both compounds, the crystal structure is stabilized by inter­molecular C—H⋯O hydrogen bonds (Tables 1 ▸ and 2 ▸). In (I), the C—H⋯O hydrogen bonds link adjacent mol­ecules, forming chains propagating along the *b*-axis direction. The chains are linked by C—H⋯Cl hydrogen bonds, forming layers parallel to the (10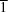) plane; see Table 1 ▸ and Fig. 6 ▸. Within the layers there are C—H⋯π inter­actions present (Table 1 ▸).

**Figure 7:**
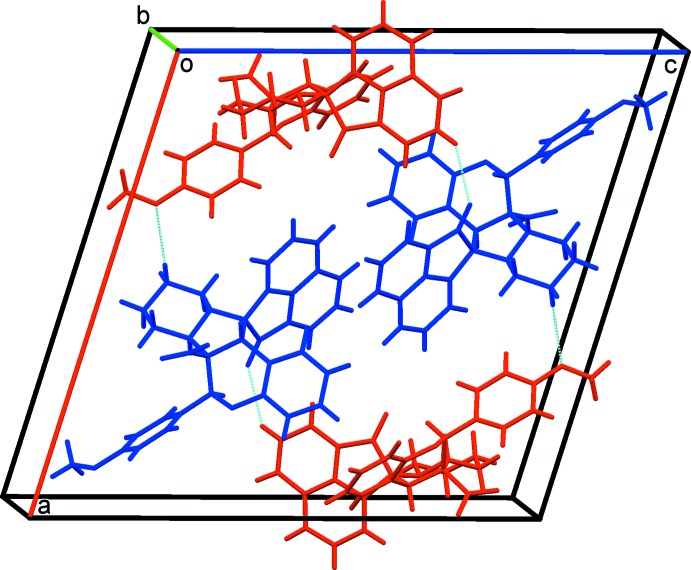
A partial view along the *b* axis of the crystal packing of compound (II). The *A* (blue) and *B* (red) mol­ecules are linked *via* C—H⋯O hydrogen bonds (dashed lines; see Table 2 ▸).

**Figure 8:**
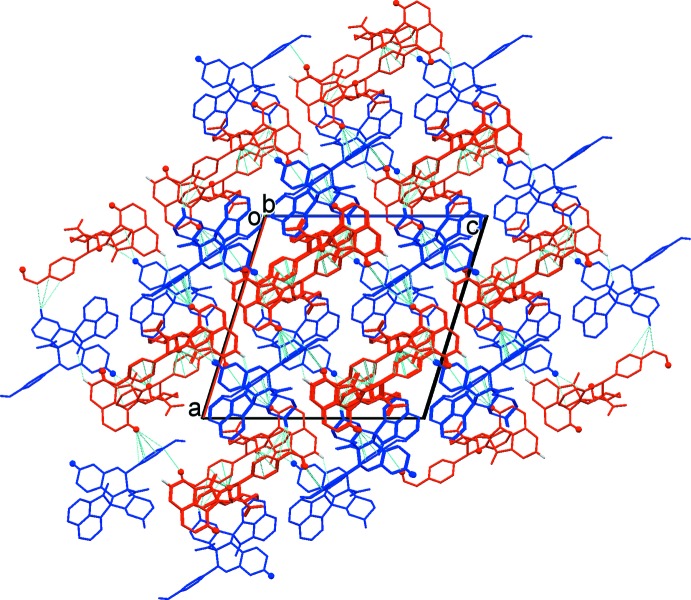
A view along the *b* axis of the crystal packing of compound (II). The *A* (blue) and *B* (red) mol­ecules are linked *via* C—H⋯O hydrogen bonds and C—H⋯π inter­actions (dashed lines; see Table 2 ▸). For clarity, H atoms not involved in the various inter­molecular inter­actions have been omitted.

In compound (II), the inter­linking of *A* and *B* mol­ecules *via* C—H⋯O hydrogen bonds generates four-membered units (Table 2 ▸ and Fig. 7 ▸). These are linked by C—H⋯π inter­actions, forming a three-dimensional supra­molecular structure (Table 2 ▸ and Fig. 8 ▸).

## Database survey   

A search of the Cambridge Structural Database (CSD, Version 5.39, August 2018; Groom *et al.*, 2016 ▸) for the 6′-(4-phen­yl)-6a′-hexa­hydro-2*H*,6′*H*,6b′*H*-spiro­[benzo­pyrano[3,4-*a*]indolizin]-2-one skeleton yielded two hits: namely 6-(4-meth­oxy­phen­yl)-6a-nitro-6,6a,6b,7,8,9,10,12a-octa­hydro­spiro­[chromeno[3,4-*a*]indolizine-12,3-indolin]-2-one (CSD refcode AFONEQ; Devi *et al.*, 2013*a* ▸) and 6-(4-meth­oxy­phen­yl)-6a-nitro-6,6a,6b,7,8,9,10,12a-octa­hydro­spiro­[chromeno[3,4-*a*]indolizine-12,3-indolin]-2-one (FIDCOM; Devi *et al.*, 2013*b* ▸).

In both compounds, the piperidine ring has a chair conformation, as do the title compounds. In AFONEQ, the pyran ring has a envelope conformation, as do the title compounds, while in FIDCOM the pyran ring has a planar conformation. In these two compounds, the pyrrolidine ring adopts an envelope conformation, while in the title compounds these rings have twisted conformations. The bond lengths and bond angles are very similar to those reported here for the title compounds.

## Synthesis and crystallization   

To a solution of ace­naphtho­quinone (1.0 mmol) and piperidine-2-carb­oxy­lic acid (1.5 mmol) in dry toluene, was added 2-(4-chloro­phen­yl)-3-nitro-2*H*-chromene (1 mmol) for (I), and 2-(4-meth­oxy­phen­yl)-3-nitro-2*H*-chromene (1 mmol) for (II), under a nitro­gen atmosphere. The solutions were refluxed for 18 h in a Dean–Stark apparatus to give the cyclo­adducts. After completion of the reactions as indicated by TLC, the solvent was evaporated under reduced pressure. The crude products obtained were purified by column chromatography using hexa­ne/EtOAc (8:2) as eluent (yield 89%). Colourless block-like crystals of the title compounds, suitable for X-ray diffraction analysis, were obtained by slow evaporation of solutions in ethanol.

## Refinement   

Crystal data, data collection and structure refinement details are summarized in Table 3 ▸. For both compounds the H atoms were positioned geometrically and constrained to ride on their parent atoms: C—H = 0.93–0.98 Å with *U*_iso_(H) = 1.5*U*_eq_(C-meth­yl) and 1.2*U*_eq_(C) for other H atoms. In compound (I) the nitro group oxygen atoms, O3 and O4, are disordered over two positions with a refined occupancy ratio of 0.54 (3):0.46 (3). In compound (II), the meth­oxy­benzene group of mol­ecule *B* is disordered, as detectable from the large displacement parameters for the C and O atoms and short C—C and C—O bond lengths. This disorder over two positions was modelled and the site occupancies refined to 0.739 (5) and 0.261 (5). The geometry was regularized by soft restraints.

## Supplementary Material

Crystal structure: contains datablock(s) global, I, II. DOI: 10.1107/S2056989019000422/su5472sup1.cif

Supporting information file. DOI: 10.1107/S2056989019000422/su5472Isup2.cml

Supporting information file. DOI: 10.1107/S2056989019000422/su5472IIsup3.cml

Puckering and asymmety parameters. DOI: 10.1107/S2056989019000422/su5472sup4.pdf

CCDC references: 1024915, 1024235

Additional supporting information:  crystallographic information; 3D view; checkCIF report

## Figures and Tables

**Figure 3:**
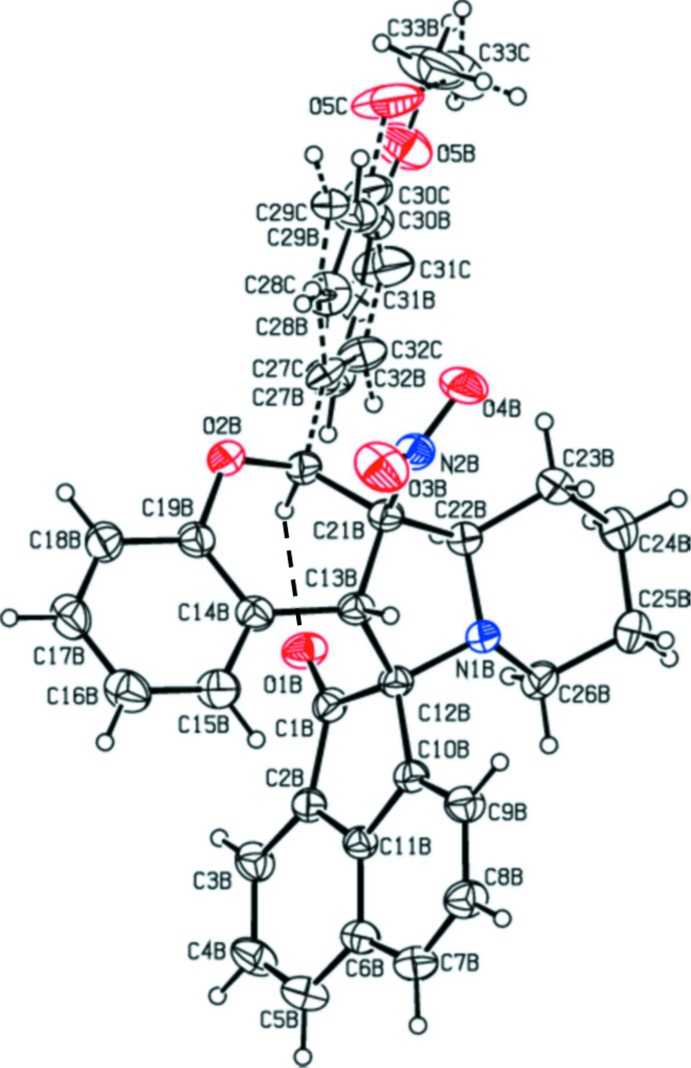
View of the mol­ecular structure of mol­ecule *B* of compound (II), with the atom labelling. Displacement ellipsoids are drawn at the 30% probability level. The intra­molecular C—H⋯O hydrogen bond (see Table 2 ▸) is shown as a dashed line.

**Table 1:** Hydrogen-bond geometry (Å, °) for (I) *Cg*1 is the centroid of the C14–C19 ring.

*D*—H⋯*A*	*D*—H	H⋯*A*	*D*⋯*A*	*D*—H⋯*A*
C20—H20⋯O1	0.98	2.46	3.284 (3)	142
C4—H4⋯O4^i^	0.93	2.59	3.481 (8)	160
C16—H16⋯Cl1^ii^	0.93	2.79	3.459 (2)	130
C9—H9⋯*Cg*1^iii^	0.93	2.88	3.607 (3)	136

Symmetry codes: (i) 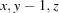; (ii) 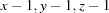; (iii) 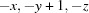.

**Table 2:** Hydrogen-bond geometry (Å, °) for (II) *Cg*1, *Cg*2, *Cg*3 and *Cg*4 are the centroids of rings C27*A*–C32*A*, C14*B*–C19*B*, C27*B*–C32*B* and C2*B*–C6*B*/C11*B*, respecively.

*D*—H⋯*A*	*D*—H	H⋯*A*	*D*⋯*A*	*D*—H⋯*A*
C20*A*—H20*A*⋯O1*A*	0.98	2.38	3.225 (2)	144
C20*B*—H20*B*⋯O1*B*	0.98	2.28	3.152 (2)	147
C4*B*—H4*B*⋯O1*A* ^i^	0.93	2.55	3.315 (2)	139
C25*A*—H25*A*⋯O5*B* ^ii^	0.97	2.53	3.233 (3)	129
C9*B*—H9*B*⋯*Cg*1^iii^	0.93	2.87	3.709 (2)	150
C17*A*—H17*A*⋯*Cg*2^iv^	0.93	2.77	3.667 (2)	161
C26*B*—H26*C*⋯*Cg*3^v^	0.97	2.88	3.575 (3)	129
C33*B*—H33*F*⋯*Cg*4^ii^	0.96	2.86	3.707 (7)	148

Symmetry codes: (i) 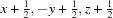; (ii) 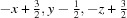; (iii) 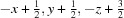; (iv) 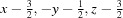; (v) 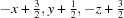.

**Table 3:** Experimental details

	(I)	(II)
Crystal data		
Chemical formula	C_32_H_25_ClN_2_O_4_	C_33_H_28_N_2_O_5_
*M*_r_	536.99	532.57
Crystal system, space group	Triclinic, *P* 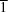	Monoclinic, *P*2_1_/*n*
Temperature (K)	293	293
*a*, *b*, *c* (Å)	10.6777 (4), 11.6095 (4), 12.6037 (8)	19.8942 (2), 13.6097 (7), 20.7822 (1)
α, β, γ (°)	98.383 (3), 105.378 (3), 115.522 (2)	90, 107.268 (3), 90
*V* (Å^3^)	1297.32 (11)	5373.2 (3)
*Z*	2	8
Radiation type	Mo *K*α	Mo *K*α
μ (mm^−1^)	0.19	0.09
Crystal size (mm)	0.20 × 0.15 × 0.10	0.30 × 0.26 × 0.20

Data collection		
Diffractometer	Bruker Kappa APEXII CCD	Bruker Kappa APEXII CCD
Absorption correction	Multi-scan (*SADABS*; Bruker, 2008 ▸)	Multi-scan (*SADABS*; Bruker, 2008 ▸)
*T*_min_, *T*_max_	0.752, 0.863	0.789, 0.846
No. of measured, independent and observed [*I* > 2σ(*I*)] reflections	19013, 5294, 3713	50874, 13194, 7862
*R*_int_	0.037	0.033
(sin θ/λ)_max_ (Å^−1^)	0.625	0.667

Refinement		
*R*[*F* ^2^ > 2σ(*F* ^2^)], *wR*(*F* ^2^), *S*	0.049, 0.150, 1.02	0.048, 0.130, 1.02
No. of reflections	5294	13194
No. of parameters	371	795
No. of restraints	2	31
H-atom treatment	H-atom parameters constrained	H-atom parameters constrained
Δρ_max_, Δρ_min_ (e Å^−3^)	0.29, −0.38	0.30, −0.20

Computer programs: *APEX2* and *SAINT* (Bruker, 2008 ▸), *SHELXS2018*/3 (Sheldrick, 2008 ▸), *SHELXL2018/3* (Sheldrick, 2015 ▸), *ORTEP-3 for Windows* and *WinGX* (Farrugia, 2012 ▸), *Mercury* (Macrae *et al.*, 2008 ▸), *PLATON* (Spek, 2009 ▸), and *publCIF* (Westrip, 2010 ▸).

## supplementary crystallographic information

### 6'-(4-Chlorophenyl)-6a'-nitro-6a',6b',7',9',10',12a'-hexahydro-2H,6'H,8'H-spiro[acenaphthylene-1,12'-chromeno[3,4-a]indolizin]-2-one (I) Crystal data

**Table:**

C_32_H_25_ClN_2_O_4_	*Z* = 2
*M**_r_* = 536.99	*F*(000) = 560
Triclinic, *P*1	*D*_x_ = 1.375 Mg m^−^^3^
*a* = 10.6777 (4) Å	Mo *K*α radiation, λ = 0.71073 Å
*b* = 11.6095 (4) Å	Cell parameters from 5294 reflections
*c* = 12.6037 (8) Å	θ = 1.8–26.4°
α = 98.383 (3)°	µ = 0.19 mm^−^^1^
β = 105.378 (3)°	*T* = 293 K
γ = 115.522 (2)°	Block, colourless
*V* = 1297.32 (11) Å^3^	0.20 × 0.15 × 0.10 mm

### 6'-(4-Chlorophenyl)-6a'-nitro-6a',6b',7',9',10',12a'-hexahydro-2H,6'H,8'H-spiro[acenaphthylene-1,12'-chromeno[3,4-a]indolizin]-2-one (I) Data collection

**Table:**

Bruker Kappa APEXII CCD diffractometer	3713 reflections with *I* > 2σ(*I*)
ω and φ scans	*R*_int_ = 0.037
Absorption correction: multi-scan (*SADABS*; Bruker, 2008)	θ_max_ = 26.4°, θ_min_ = 1.8°
*T*_min_ = 0.752, *T*_max_ = 0.863	*h* = −11→13
19013 measured reflections	*k* = −14→14
5294 independent reflections	*l* = −15→15

### 6'-(4-Chlorophenyl)-6a'-nitro-6a',6b',7',9',10',12a'-hexahydro-2H,6'H,8'H-spiro[acenaphthylene-1,12'-chromeno[3,4-a]indolizin]-2-one (I) Refinement

**Table:**

Refinement on *F*^2^	2 restraints
Least-squares matrix: full	Hydrogen site location: inferred from neighbouring sites
*R*[*F*^2^ > 2σ(*F*^2^)] = 0.049	H-atom parameters constrained
*wR*(*F*^2^) = 0.150	*w* = 1/[σ^2^(*F*_o_^2^) + (0.0789*P*)^2^ + 0.345*P*] where *P* = (*F*_o_^2^ + 2*F*_c_^2^)/3
*S* = 1.02	(Δ/σ)_max_ = 0.001
5294 reflections	Δρ_max_ = 0.29 e Å^−^^3^
371 parameters	Δρ_min_ = −0.38 e Å^−^^3^

### 6'-(4-Chlorophenyl)-6a'-nitro-6a',6b',7',9',10',12a'-hexahydro-2H,6'H,8'H-spiro[acenaphthylene-1,12'-chromeno[3,4-a]indolizin]-2-one (I) Special details

**Table:**

Geometry. All esds (except the esd in the dihedral angle between two l.s. planes) are estimated using the full covariance matrix. The cell esds are taken into account individually in the estimation of esds in distances, angles and torsion angles; correlations between esds in cell parameters are only used when they are defined by crystal symmetry. An approximate (isotropic) treatment of cell esds is used for estimating esds involving l.s. planes.

### 6'-(4-Chlorophenyl)-6a'-nitro-6a',6b',7',9',10',12a'-hexahydro-2H,6'H,8'H-spiro[acenaphthylene-1,12'-chromeno[3,4-a]indolizin]-2-one (I) Fractional atomic coordinates and isotropic or equivalent isotropic displacement parameters (Å^2^)

**Table:**

	*x*	*y*	*z*	*U*_iso_*/*U*_eq_	Occ. (<1)
C1	0.2881 (2)	0.3825 (2)	0.25371 (18)	0.0459 (5)	
C2	0.1842 (2)	0.2368 (2)	0.21187 (17)	0.0466 (5)	
C3	0.2047 (3)	0.1270 (2)	0.20568 (19)	0.0561 (6)	
H3	0.299884	0.137107	0.232341	0.067*	
C4	0.0781 (3)	−0.0002 (2)	0.1582 (2)	0.0646 (7)	
H4	0.090771	−0.074825	0.155382	0.077*	
C5	−0.0632 (3)	−0.0190 (2)	0.11575 (19)	0.0609 (6)	
H5	−0.143848	−0.105415	0.083782	0.073*	
C6	−0.0885 (3)	0.0914 (2)	0.11975 (17)	0.0492 (5)	
C7	−0.2264 (3)	0.0891 (2)	0.07867 (19)	0.0579 (6)	
H7	−0.314492	0.007943	0.041761	0.069*	
C8	−0.2302 (3)	0.2063 (2)	0.0933 (2)	0.0606 (6)	
H8	−0.322135	0.202763	0.066518	0.073*	
C9	−0.1006 (2)	0.3325 (2)	0.1472 (2)	0.0550 (6)	
H9	−0.107296	0.410328	0.157064	0.066*	
C10	0.0346 (2)	0.33842 (19)	0.18448 (17)	0.0438 (5)	
C11	0.0387 (2)	0.21777 (19)	0.17054 (16)	0.0438 (5)	
C12	0.1921 (2)	0.45489 (18)	0.24065 (17)	0.0419 (4)	
C13	0.2339 (2)	0.55885 (18)	0.17313 (16)	0.0409 (4)	
H13	0.146419	0.570080	0.144336	0.049*	
C14	0.2711 (2)	0.52501 (19)	0.06933 (17)	0.0453 (5)	
C15	0.1738 (3)	0.4053 (2)	−0.02013 (18)	0.0578 (6)	
H15	0.089528	0.341149	−0.012400	0.069*	
C16	0.2015 (3)	0.3815 (3)	−0.11943 (19)	0.0674 (7)	
H16	0.137258	0.300386	−0.177120	0.081*	
C17	0.3231 (3)	0.4764 (3)	−0.13407 (19)	0.0622 (7)	
H17	0.338960	0.460843	−0.202545	0.075*	
C18	0.4217 (3)	0.5951 (2)	−0.04682 (19)	0.0563 (6)	
H18	0.505257	0.659264	−0.055468	0.068*	
C19	0.3945 (2)	0.6173 (2)	0.05370 (17)	0.0467 (5)	
C20	0.5079 (2)	0.7327 (2)	0.25230 (17)	0.0437 (5)	
H20	0.530350	0.660589	0.262283	0.052*	
C21	0.3561 (2)	0.69182 (18)	0.26509 (16)	0.0398 (4)	
C22	0.3564 (2)	0.66574 (19)	0.38201 (17)	0.0423 (4)	
H22	0.437692	0.646499	0.411112	0.051*	
C23	0.3709 (3)	0.7696 (2)	0.48007 (18)	0.0509 (5)	
H23A	0.465123	0.851367	0.502512	0.061*	
H23B	0.290754	0.789981	0.455425	0.061*	
C24	0.3637 (3)	0.7141 (2)	0.58148 (19)	0.0601 (6)	
H24B	0.367040	0.777458	0.643088	0.072*	
H24A	0.449553	0.702064	0.610330	0.072*	
C25	0.2216 (3)	0.5812 (2)	0.5462 (2)	0.0642 (6)	
H25A	0.222340	0.544821	0.610801	0.077*	
H25B	0.136013	0.594954	0.525425	0.077*	
C26	0.2080 (3)	0.4825 (2)	0.44468 (19)	0.0567 (6)	
H26A	0.287900	0.461395	0.467360	0.068*	
H26B	0.113644	0.400231	0.419912	0.068*	
C27	0.6420 (2)	0.8610 (2)	0.33494 (17)	0.0445 (5)	
C28	0.6967 (2)	0.9762 (2)	0.30247 (18)	0.0528 (5)	
H28	0.649586	0.974101	0.228077	0.063*	
C29	0.8183 (3)	1.0935 (2)	0.37688 (19)	0.0563 (6)	
H29	0.854267	1.169595	0.352950	0.068*	
C30	0.8866 (2)	1.0972 (2)	0.48772 (19)	0.0540 (5)	
C31	0.8373 (3)	0.9844 (3)	0.5221 (2)	0.0630 (6)	
H31	0.884924	0.987365	0.596696	0.076*	
C32	0.7169 (2)	0.8664 (2)	0.4457 (2)	0.0571 (6)	
H32	0.685213	0.789244	0.468568	0.069*	
N2	0.3151 (2)	0.80137 (17)	0.24890 (16)	0.0485 (4)	
N1	0.21621 (18)	0.54109 (15)	0.34986 (14)	0.0438 (4)	
O1	0.42335 (18)	0.43921 (15)	0.29281 (15)	0.0614 (4)	
O2	0.49612 (16)	0.73855 (14)	0.13750 (12)	0.0521 (4)	
Cl1	1.03556 (8)	1.24711 (7)	0.58385 (6)	0.0799 (2)	
O3	0.3964 (11)	0.9095 (5)	0.3177 (6)	0.0596 (18)	0.54 (3)
O4	0.2186 (17)	0.7792 (7)	0.1611 (8)	0.076 (3)	0.54 (3)
O3'	0.4053 (12)	0.9176 (7)	0.281 (2)	0.107 (5)	0.46 (3)
O4'	0.1793 (6)	0.7649 (6)	0.214 (2)	0.085 (5)	0.46 (3)

### 6'-(4-Chlorophenyl)-6a'-nitro-6a',6b',7',9',10',12a'-hexahydro-2H,6'H,8'H-spiro[acenaphthylene-1,12'-chromeno[3,4-a]indolizin]-2-one (I) Atomic displacement parameters (Å^2^)

**Table:**

	*U*^11^	*U*^22^	*U*^33^	*U*^12^	*U*^13^	*U*^23^
C1	0.0514 (13)	0.0431 (10)	0.0460 (11)	0.0284 (10)	0.0115 (9)	0.0154 (9)
C2	0.0584 (13)	0.0423 (10)	0.0409 (10)	0.0299 (10)	0.0114 (9)	0.0137 (8)
C3	0.0726 (15)	0.0511 (12)	0.0515 (12)	0.0417 (12)	0.0125 (11)	0.0169 (10)
C4	0.096 (2)	0.0437 (12)	0.0574 (14)	0.0436 (13)	0.0162 (13)	0.0155 (10)
C5	0.0838 (18)	0.0366 (11)	0.0481 (12)	0.0257 (11)	0.0110 (12)	0.0115 (9)
C6	0.0636 (14)	0.0391 (10)	0.0381 (10)	0.0229 (10)	0.0125 (10)	0.0128 (8)
C7	0.0543 (14)	0.0459 (12)	0.0502 (12)	0.0130 (10)	0.0050 (10)	0.0155 (10)
C8	0.0460 (13)	0.0537 (13)	0.0694 (15)	0.0213 (11)	0.0064 (11)	0.0221 (11)
C9	0.0512 (13)	0.0452 (11)	0.0678 (14)	0.0268 (10)	0.0129 (11)	0.0209 (10)
C10	0.0474 (12)	0.0372 (10)	0.0453 (11)	0.0226 (9)	0.0103 (9)	0.0147 (8)
C11	0.0536 (12)	0.0387 (10)	0.0375 (10)	0.0245 (9)	0.0103 (9)	0.0135 (8)
C12	0.0447 (11)	0.0374 (10)	0.0435 (10)	0.0236 (9)	0.0100 (9)	0.0126 (8)
C13	0.0417 (11)	0.0371 (10)	0.0438 (10)	0.0233 (9)	0.0079 (8)	0.0135 (8)
C14	0.0540 (12)	0.0423 (10)	0.0390 (10)	0.0294 (10)	0.0056 (9)	0.0128 (8)
C15	0.0722 (16)	0.0471 (12)	0.0417 (12)	0.0277 (11)	0.0061 (11)	0.0124 (9)
C16	0.098 (2)	0.0561 (14)	0.0375 (11)	0.0412 (14)	0.0056 (12)	0.0090 (10)
C17	0.0880 (18)	0.0741 (16)	0.0391 (11)	0.0563 (15)	0.0170 (12)	0.0155 (11)
C18	0.0625 (14)	0.0684 (14)	0.0487 (12)	0.0430 (12)	0.0169 (11)	0.0183 (11)
C19	0.0539 (13)	0.0490 (11)	0.0413 (11)	0.0333 (10)	0.0104 (9)	0.0122 (9)
C20	0.0449 (11)	0.0450 (10)	0.0418 (10)	0.0243 (9)	0.0134 (9)	0.0124 (9)
C21	0.0430 (11)	0.0336 (9)	0.0453 (10)	0.0234 (8)	0.0116 (9)	0.0119 (8)
C22	0.0420 (11)	0.0398 (10)	0.0450 (11)	0.0218 (9)	0.0122 (9)	0.0138 (8)
C23	0.0495 (12)	0.0435 (11)	0.0521 (12)	0.0199 (10)	0.0163 (10)	0.0075 (9)
C24	0.0644 (15)	0.0601 (14)	0.0450 (12)	0.0274 (12)	0.0143 (11)	0.0074 (10)
C25	0.0713 (16)	0.0615 (14)	0.0509 (13)	0.0249 (13)	0.0226 (12)	0.0175 (11)
C26	0.0623 (14)	0.0477 (12)	0.0529 (13)	0.0207 (11)	0.0190 (11)	0.0199 (10)
C27	0.0406 (11)	0.0495 (11)	0.0437 (11)	0.0228 (9)	0.0149 (9)	0.0139 (9)
C28	0.0537 (13)	0.0553 (13)	0.0396 (11)	0.0206 (11)	0.0125 (10)	0.0161 (10)
C29	0.0571 (14)	0.0511 (12)	0.0470 (12)	0.0170 (11)	0.0154 (10)	0.0155 (10)
C30	0.0461 (12)	0.0584 (13)	0.0435 (11)	0.0181 (10)	0.0132 (10)	0.0080 (10)
C31	0.0509 (13)	0.0762 (16)	0.0445 (12)	0.0220 (12)	0.0063 (10)	0.0220 (12)
C32	0.0483 (13)	0.0594 (13)	0.0568 (13)	0.0218 (11)	0.0122 (10)	0.0274 (11)
N2	0.0535 (11)	0.0410 (10)	0.0576 (11)	0.0287 (9)	0.0184 (9)	0.0182 (9)
N1	0.0466 (10)	0.0367 (8)	0.0452 (9)	0.0198 (7)	0.0136 (8)	0.0127 (7)
O1	0.0505 (10)	0.0527 (9)	0.0795 (11)	0.0307 (8)	0.0117 (8)	0.0208 (8)
O2	0.0527 (9)	0.0507 (8)	0.0434 (8)	0.0196 (7)	0.0161 (7)	0.0099 (6)
Cl1	0.0707 (4)	0.0686 (4)	0.0534 (4)	0.0104 (3)	0.0042 (3)	0.0021 (3)
O3	0.073 (4)	0.026 (2)	0.066 (4)	0.021 (2)	0.014 (2)	0.005 (2)
O4	0.095 (5)	0.056 (2)	0.065 (3)	0.049 (3)	−0.008 (3)	0.015 (2)
O3'	0.064 (4)	0.044 (3)	0.224 (13)	0.030 (3)	0.057 (7)	0.057 (5)
O4'	0.059 (3)	0.058 (3)	0.131 (11)	0.039 (2)	0.008 (3)	0.023 (4)

### 6'-(4-Chlorophenyl)-6a'-nitro-6a',6b',7',9',10',12a'-hexahydro-2H,6'H,8'H-spiro[acenaphthylene-1,12'-chromeno[3,4-a]indolizin]-2-one (I) Geometric parameters (Å, º)

**Table:**

C1—O1	1.213 (3)	C20—O2	1.433 (2)
C1—C2	1.477 (3)	C20—C27	1.507 (3)
C1—C12	1.574 (3)	C20—C21	1.542 (3)
C2—C3	1.378 (3)	C20—H20	0.9800
C2—C11	1.406 (3)	C21—N2	1.535 (2)
C3—C4	1.399 (3)	C21—C22	1.547 (3)
C3—H3	0.9300	C22—N1	1.463 (2)
C4—C5	1.365 (4)	C22—C23	1.519 (3)
C4—H4	0.9300	C22—H22	0.9800
C5—C6	1.416 (3)	C23—C24	1.518 (3)
C5—H5	0.9300	C23—H23A	0.9700
C6—C11	1.398 (3)	C23—H23B	0.9700
C6—C7	1.412 (3)	C24—C25	1.519 (3)
C7—C8	1.366 (3)	C24—H24B	0.9700
C7—H7	0.9300	C24—H24A	0.9700
C8—C9	1.409 (3)	C25—C26	1.517 (3)
C8—H8	0.9300	C25—H25A	0.9700
C9—C10	1.362 (3)	C25—H25B	0.9700
C9—H9	0.9300	C26—N1	1.463 (3)
C10—C11	1.407 (3)	C26—H26A	0.9700
C10—C12	1.512 (3)	C26—H26B	0.9700
C12—N1	1.466 (3)	C27—C28	1.383 (3)
C12—C13	1.555 (3)	C27—C32	1.390 (3)
C13—C14	1.511 (3)	C28—C29	1.371 (3)
C13—C21	1.539 (3)	C28—H28	0.9300
C13—H13	0.9800	C29—C30	1.379 (3)
C14—C19	1.380 (3)	C29—H29	0.9300
C14—C15	1.400 (3)	C30—C31	1.368 (3)
C15—C16	1.377 (3)	C30—Cl1	1.734 (2)
C15—H15	0.9300	C31—C32	1.378 (3)
C16—C17	1.374 (4)	C31—H31	0.9300
C16—H16	0.9300	C32—H32	0.9300
C17—C18	1.382 (3)	N2—O3'	1.194 (6)
C17—H17	0.9300	N2—O3	1.194 (5)
C18—C19	1.386 (3)	N2—O4	1.199 (4)
C18—H18	0.9300	N2—O4'	1.247 (5)
C19—O2	1.385 (2)		
			
O1—C1—C2	127.43 (19)	C27—C20—H20	106.7
O1—C1—C12	124.78 (18)	C21—C20—H20	106.7
C2—C1—C12	107.79 (17)	N2—C21—C13	108.98 (15)
C3—C2—C11	119.4 (2)	N2—C21—C20	108.70 (15)
C3—C2—C1	133.3 (2)	C13—C21—C20	110.44 (15)
C11—C2—C1	107.33 (17)	N2—C21—C22	111.16 (15)
C2—C3—C4	118.1 (2)	C13—C21—C22	105.13 (15)
C2—C3—H3	120.9	C20—C21—C22	112.37 (15)
C4—C3—H3	120.9	N1—C22—C23	110.13 (17)
C5—C4—C3	122.6 (2)	N1—C22—C21	103.32 (15)
C5—C4—H4	118.7	C23—C22—C21	120.98 (16)
C3—C4—H4	118.7	N1—C22—H22	107.2
C4—C5—C6	120.9 (2)	C23—C22—H22	107.2
C4—C5—H5	119.5	C21—C22—H22	107.2
C6—C5—H5	119.5	C24—C23—C22	108.70 (18)
C11—C6—C7	116.29 (18)	C24—C23—H23A	110.0
C11—C6—C5	115.8 (2)	C22—C23—H23A	110.0
C7—C6—C5	127.9 (2)	C24—C23—H23B	110.0
C8—C7—C6	119.8 (2)	C22—C23—H23B	110.0
C8—C7—H7	120.1	H23A—C23—H23B	108.3
C6—C7—H7	120.1	C23—C24—C25	110.87 (19)
C7—C8—C9	122.8 (2)	C23—C24—H24B	109.5
C7—C8—H8	118.6	C25—C24—H24B	109.5
C9—C8—H8	118.6	C23—C24—H24A	109.5
C10—C9—C8	118.8 (2)	C25—C24—H24A	109.5
C10—C9—H9	120.6	H24B—C24—H24A	108.1
C8—C9—H9	120.6	C26—C25—C24	110.8 (2)
C9—C10—C11	118.46 (19)	C26—C25—H25A	109.5
C9—C10—C12	132.14 (18)	C24—C25—H25A	109.5
C11—C10—C12	109.40 (17)	C26—C25—H25B	109.5
C6—C11—C2	123.11 (18)	C24—C25—H25B	109.5
C6—C11—C10	123.72 (19)	H25A—C25—H25B	108.1
C2—C11—C10	113.16 (18)	N1—C26—C25	109.29 (18)
N1—C12—C10	112.51 (17)	N1—C26—H26A	109.8
N1—C12—C13	100.06 (14)	C25—C26—H26A	109.8
C10—C12—C13	114.76 (15)	N1—C26—H26B	109.8
N1—C12—C1	114.02 (15)	C25—C26—H26B	109.8
C10—C12—C1	102.23 (15)	H26A—C26—H26B	108.3
C13—C12—C1	113.83 (16)	C28—C27—C32	117.78 (19)
C14—C13—C21	114.28 (17)	C28—C27—C20	121.29 (18)
C14—C13—C12	118.24 (15)	C32—C27—C20	120.91 (19)
C21—C13—C12	105.24 (15)	C29—C28—C27	121.8 (2)
C14—C13—H13	106.1	C29—C28—H28	119.1
C21—C13—H13	106.1	C27—C28—H28	119.1
C12—C13—H13	106.1	C28—C29—C30	119.0 (2)
C19—C14—C15	117.5 (2)	C28—C29—H29	120.5
C19—C14—C13	120.89 (17)	C30—C29—H29	120.5
C15—C14—C13	121.3 (2)	C31—C30—C29	120.7 (2)
C16—C15—C14	120.8 (2)	C31—C30—Cl1	120.15 (18)
C16—C15—H15	119.6	C29—C30—Cl1	119.15 (18)
C14—C15—H15	119.6	C30—C31—C32	119.7 (2)
C17—C16—C15	120.6 (2)	C30—C31—H31	120.2
C17—C16—H16	119.7	C32—C31—H31	120.2
C15—C16—H16	119.7	C31—C32—C27	120.9 (2)
C16—C17—C18	119.9 (2)	C31—C32—H32	119.5
C16—C17—H17	120.1	C27—C32—H32	119.5
C18—C17—H17	120.1	O3—N2—O4	123.8 (5)
C17—C18—C19	119.1 (2)	O3'—N2—O4'	120.1 (7)
C17—C18—H18	120.4	O3'—N2—C21	123.1 (7)
C19—C18—H18	120.4	O3—N2—C21	116.7 (5)
C14—C19—O2	121.30 (18)	O4—N2—C21	118.5 (3)
C14—C19—C18	122.1 (2)	O4'—N2—C21	116.0 (4)
O2—C19—C18	116.6 (2)	C22—N1—C26	112.40 (16)
O2—C20—C27	107.76 (16)	C22—N1—C12	107.98 (16)
O2—C20—C21	110.04 (16)	C26—N1—C12	116.80 (15)
C27—C20—C21	118.30 (16)	C19—O2—C20	113.34 (15)
O2—C20—H20	106.7		
			
O1—C1—C2—C3	1.2 (4)	C14—C13—C21—C20	−21.1 (2)
C12—C1—C2—C3	−178.0 (2)	C12—C13—C21—C20	110.28 (17)
O1—C1—C2—C11	−177.8 (2)	C14—C13—C21—C22	−142.51 (16)
C12—C1—C2—C11	3.0 (2)	C12—C13—C21—C22	−11.14 (19)
C11—C2—C3—C4	−0.1 (3)	O2—C20—C21—N2	−64.05 (19)
C1—C2—C3—C4	−179.0 (2)	C27—C20—C21—N2	60.4 (2)
C2—C3—C4—C5	1.5 (4)	O2—C20—C21—C13	55.46 (19)
C3—C4—C5—C6	−1.1 (4)	C27—C20—C21—C13	179.88 (16)
C4—C5—C6—C11	−0.8 (3)	O2—C20—C21—C22	172.49 (15)
C4—C5—C6—C7	179.1 (2)	C27—C20—C21—C22	−63.1 (2)
C11—C6—C7—C8	−2.2 (3)	N2—C21—C22—N1	103.19 (17)
C5—C6—C7—C8	177.9 (2)	C13—C21—C22—N1	−14.58 (18)
C6—C7—C8—C9	0.8 (4)	C20—C21—C22—N1	−134.74 (15)
C7—C8—C9—C10	1.3 (4)	N2—C21—C22—C23	−20.5 (2)
C8—C9—C10—C11	−1.8 (3)	C13—C21—C22—C23	−138.27 (18)
C8—C9—C10—C12	177.6 (2)	C20—C21—C22—C23	101.6 (2)
C7—C6—C11—C2	−177.70 (19)	N1—C22—C23—C24	58.2 (2)
C5—C6—C11—C2	2.3 (3)	C21—C22—C23—C24	178.58 (18)
C7—C6—C11—C10	1.6 (3)	C22—C23—C24—C25	−55.8 (3)
C5—C6—C11—C10	−178.4 (2)	C23—C24—C25—C26	55.5 (3)
C3—C2—C11—C6	−1.8 (3)	C24—C25—C26—N1	−55.7 (3)
C1—C2—C11—C6	177.29 (18)	O2—C20—C27—C28	23.7 (3)
C3—C2—C11—C10	178.78 (19)	C21—C20—C27—C28	−101.8 (2)
C1—C2—C11—C10	−2.1 (2)	O2—C20—C27—C32	−154.8 (2)
C9—C10—C11—C6	0.4 (3)	C21—C20—C27—C32	79.7 (3)
C12—C10—C11—C6	−179.14 (18)	C32—C27—C28—C29	−1.6 (3)
C9—C10—C11—C2	179.76 (19)	C20—C27—C28—C29	179.9 (2)
C12—C10—C11—C2	0.2 (2)	C27—C28—C29—C30	−1.0 (4)
C9—C10—C12—N1	59.4 (3)	C28—C29—C30—C31	2.3 (4)
C11—C10—C12—N1	−121.17 (18)	C28—C29—C30—Cl1	−177.30 (18)
C9—C10—C12—C13	−54.1 (3)	C29—C30—C31—C32	−0.9 (4)
C11—C10—C12—C13	125.30 (18)	Cl1—C30—C31—C32	178.65 (19)
C9—C10—C12—C1	−177.9 (2)	C30—C31—C32—C27	−1.7 (4)
C11—C10—C12—C1	1.6 (2)	C28—C27—C32—C31	2.9 (3)
O1—C1—C12—N1	−60.3 (3)	C20—C27—C32—C31	−178.5 (2)
C2—C1—C12—N1	118.94 (18)	C13—C21—N2—O3'	−156.1 (16)
O1—C1—C12—C10	178.0 (2)	C20—C21—N2—O3'	−35.7 (16)
C2—C1—C12—C10	−2.8 (2)	C22—C21—N2—O3'	88.5 (16)
O1—C1—C12—C13	53.7 (3)	C13—C21—N2—O3	176.6 (5)
C2—C1—C12—C13	−127.12 (18)	C20—C21—N2—O3	−62.9 (6)
N1—C12—C13—C14	161.39 (17)	C22—C21—N2—O3	61.2 (6)
C10—C12—C13—C14	−77.9 (2)	C13—C21—N2—O4	−13.9 (11)
C1—C12—C13—C14	39.4 (2)	C20—C21—N2—O4	106.6 (11)
N1—C12—C13—C21	32.34 (18)	C22—C21—N2—O4	−129.3 (11)
C10—C12—C13—C21	153.01 (16)	C13—C21—N2—O4'	33.7 (14)
C1—C12—C13—C21	−89.68 (18)	C20—C21—N2—O4'	154.1 (14)
C21—C13—C14—C19	−9.2 (2)	C22—C21—N2—O4'	−81.7 (14)
C12—C13—C14—C19	−133.89 (19)	C23—C22—N1—C26	−61.6 (2)
C21—C13—C14—C15	177.82 (17)	C21—C22—N1—C26	167.85 (16)
C12—C13—C14—C15	53.1 (2)	C23—C22—N1—C12	168.13 (16)
C19—C14—C15—C16	0.2 (3)	C21—C22—N1—C12	37.57 (18)
C13—C14—C15—C16	173.4 (2)	C25—C26—N1—C22	59.5 (2)
C14—C15—C16—C17	−1.8 (4)	C25—C26—N1—C12	−174.91 (18)
C15—C16—C17—C18	2.3 (4)	C10—C12—N1—C22	−166.20 (15)
C16—C17—C18—C19	−1.1 (3)	C13—C12—N1—C22	−43.92 (18)
C15—C14—C19—O2	179.66 (17)	C1—C12—N1—C22	77.97 (19)
C13—C14—C19—O2	6.4 (3)	C10—C12—N1—C26	66.0 (2)
C15—C14—C19—C18	1.0 (3)	C13—C12—N1—C26	−171.71 (17)
C13—C14—C19—C18	−172.22 (18)	C1—C12—N1—C26	−49.8 (2)
C17—C18—C19—C14	−0.6 (3)	C14—C19—O2—C20	29.9 (2)
C17—C18—C19—O2	−179.25 (18)	C18—C19—O2—C20	−151.41 (18)
C14—C13—C21—N2	98.26 (18)	C27—C20—O2—C19	168.71 (16)
C12—C13—C21—N2	−130.38 (16)	C21—C20—O2—C19	−61.0 (2)

### 6'-(4-Chlorophenyl)-6a'-nitro-6a',6b',7',9',10',12a'-hexahydro-2H,6'H,8'H-spiro[acenaphthylene-1,12'-chromeno[3,4-a]indolizin]-2-one (I) Hydrogen-bond geometry (Å, º)

*Cg*1 is the centroid of the C14–C19 ring.

**Table:**

*D*—H···*A*	*D*—H	H···*A*	*D*···*A*	*D*—H···*A*
C20—H20···O1	0.98	2.46	3.284 (3)	142
C4—H4···O4^i^	0.93	2.59	3.481 (8)	160
C16—H16···Cl1^ii^	0.93	2.79	3.459 (2)	130
C9—H9···*Cg*1^iii^	0.93	2.88	3.607 (3)	136

Symmetry codes: (i) *x*, *y*−1, *z*; (ii) *x*−1, *y*−1, *z*−1; (iii) −*x*, −*y*+1, −*z*.

### 6'-(4-Methoxyphenyl)-6a'-nitro-6a',6b',7',9',10',12a'-hexahydro-2H,6'H,8'H-spiro[acenaphthylene-1,12'-chromeno[3,4-a]indolizin]-2-one (II) Crystal data

**Table:**

C_33_H_28_N_2_O_5_	*F*(000) = 2240
*M**_r_* = 532.57	*D*_x_ = 1.317 Mg m^−^^3^
Monoclinic, *P*2_1_/*n*	Mo *K*α radiation, λ = 0.71073 Å
*a* = 19.8942 (2) Å	Cell parameters from 13194 reflections
*b* = 13.6097 (7) Å	θ = 1.8–28.3°
*c* = 20.7822 (1) Å	µ = 0.09 mm^−^^1^
β = 107.268 (3)°	*T* = 293 K
*V* = 5373.2 (3) Å^3^	Block, colourless
*Z* = 8	0.30 × 0.26 × 0.20 mm

### 6'-(4-Methoxyphenyl)-6a'-nitro-6a',6b',7',9',10',12a'-hexahydro-2H,6'H,8'H-spiro[acenaphthylene-1,12'-chromeno[3,4-a]indolizin]-2-one (II) Data collection

**Table:**

Bruker Kappa APEXII CCD diffractometer	7862 reflections with *I* > 2σ(*I*)
ω and φ scans	*R*_int_ = 0.033
Absorption correction: multi-scan (*SADABS*; Bruker, 2008)	θ_max_ = 28.3°, θ_min_ = 1.8°
*T*_min_ = 0.789, *T*_max_ = 0.846	*h* = −26→26
50874 measured reflections	*k* = −13→17
13194 independent reflections	*l* = −27→22

### 6'-(4-Methoxyphenyl)-6a'-nitro-6a',6b',7',9',10',12a'-hexahydro-2H,6'H,8'H-spiro[acenaphthylene-1,12'-chromeno[3,4-a]indolizin]-2-one (II) Refinement

**Table:**

Refinement on *F*^2^	31 restraints
Least-squares matrix: full	Hydrogen site location: inferred from neighbouring sites
*R*[*F*^2^ > 2σ(*F*^2^)] = 0.048	H-atom parameters constrained
*wR*(*F*^2^) = 0.130	*w* = 1/[σ^2^(*F*_o_^2^) + (0.049*P*)^2^ + 1.2075*P*] where *P* = (*F*_o_^2^ + 2*F*_c_^2^)/3
*S* = 1.02	(Δ/σ)_max_ = 0.001
13194 reflections	Δρ_max_ = 0.30 e Å^−^^3^
795 parameters	Δρ_min_ = −0.20 e Å^−^^3^

### 6'-(4-Methoxyphenyl)-6a'-nitro-6a',6b',7',9',10',12a'-hexahydro-2H,6'H,8'H-spiro[acenaphthylene-1,12'-chromeno[3,4-a]indolizin]-2-one (II) Special details

**Table:**

Geometry. All esds (except the esd in the dihedral angle between two l.s. planes) are estimated using the full covariance matrix. The cell esds are taken into account individually in the estimation of esds in distances, angles and torsion angles; correlations between esds in cell parameters are only used when they are defined by crystal symmetry. An approximate (isotropic) treatment of cell esds is used for estimating esds involving l.s. planes.

### 6'-(4-Methoxyphenyl)-6a'-nitro-6a',6b',7',9',10',12a'-hexahydro-2H,6'H,8'H-spiro[acenaphthylene-1,12'-chromeno[3,4-a]indolizin]-2-one (II) Fractional atomic coordinates and isotropic or equivalent isotropic displacement parameters (Å^2^)

**Table:**

	*x*	*y*	*z*	*U*_iso_*/*U*_eq_	Occ. (<1)
C33A	0.11183 (18)	−0.0458 (3)	0.94638 (17)	0.1429 (14)	
H33A	0.091028	−0.100767	0.918673	0.214*	
H33B	0.086829	−0.033549	0.978617	0.214*	
H33C	0.160207	−0.060057	0.969642	0.214*	
C1A	0.37825 (8)	−0.08129 (11)	0.65483 (8)	0.0434 (4)	
C1B	0.66638 (8)	0.51698 (11)	0.87412 (8)	0.0426 (4)	
C2A	0.41462 (8)	−0.11334 (12)	0.60633 (8)	0.0453 (4)	
C2B	0.65231 (8)	0.54727 (11)	0.93711 (8)	0.0422 (4)	
C3A	0.39800 (10)	−0.18088 (14)	0.55500 (9)	0.0600 (5)	
H3A	0.356240	−0.216408	0.545117	0.072*	
C3B	0.69487 (9)	0.56035 (13)	1.00181 (8)	0.0541 (4)	
H3B	0.742803	0.546846	1.013331	0.065*	
C4A	0.44547 (13)	−0.19487 (17)	0.51797 (10)	0.0736 (6)	
H4A	0.434672	−0.240401	0.483024	0.088*	
C4B	0.66398 (10)	0.59473 (16)	1.05019 (9)	0.0650 (5)	
H4B	0.692362	0.604339	1.094171	0.078*	
C5A	0.50731 (12)	−0.14379 (17)	0.53144 (10)	0.0706 (6)	
H5A	0.537090	−0.154749	0.505112	0.085*	
C5B	0.59352 (10)	0.61455 (15)	1.03476 (9)	0.0620 (5)	
H5B	0.575339	0.637806	1.068246	0.074*	
C6A	0.52695 (9)	−0.07461 (14)	0.58457 (9)	0.0535 (4)	
C6B	0.54800 (9)	0.60039 (12)	0.96908 (8)	0.0477 (4)	
C7A	0.58937 (10)	−0.01846 (16)	0.60677 (11)	0.0653 (5)	
H7A	0.623616	−0.023772	0.584710	0.078*	
C7B	0.47451 (9)	0.61578 (13)	0.94512 (10)	0.0571 (5)	
H7B	0.450423	0.637686	0.974494	0.069*	
C8A	0.59949 (9)	0.04308 (15)	0.66006 (11)	0.0647 (5)	
H8A	0.641245	0.078607	0.673949	0.078*	
C8B	0.43870 (9)	0.59871 (14)	0.87922 (9)	0.0578 (5)	
H8B	0.390427	0.610009	0.864580	0.069*	
C9A	0.54936 (8)	0.05575 (13)	0.69556 (9)	0.0539 (4)	
H9A	0.557795	0.098970	0.731748	0.065*	
C9B	0.47216 (9)	0.56451 (13)	0.83236 (9)	0.0524 (4)	
H9B	0.446413	0.553579	0.787657	0.063*	
C10A	0.48837 (8)	0.00295 (11)	0.67535 (8)	0.0421 (4)	
C10B	0.54287 (8)	0.54781 (11)	0.85382 (8)	0.0419 (4)	
C11A	0.47845 (8)	−0.06154 (12)	0.62081 (8)	0.0429 (4)	
C11B	0.57983 (8)	0.56623 (11)	0.92149 (8)	0.0406 (3)	
C12A	0.42520 (7)	−0.00163 (11)	0.70198 (8)	0.0392 (3)	
C12B	0.59354 (8)	0.51258 (11)	0.81684 (8)	0.0398 (3)	
C13A	0.38740 (8)	0.09837 (11)	0.70197 (8)	0.0412 (3)	
H13A	0.424361	0.148054	0.717293	0.049*	
C13B	0.57563 (8)	0.40974 (11)	0.78342 (8)	0.0424 (4)	
H13B	0.524655	0.408968	0.761728	0.051*	
C14A	0.33912 (8)	0.13395 (12)	0.63518 (8)	0.0467 (4)	
C14B	0.59240 (8)	0.31992 (12)	0.82777 (8)	0.0457 (4)	
C15A	0.36298 (10)	0.14494 (15)	0.57927 (10)	0.0666 (5)	
H15A	0.409068	0.127670	0.582403	0.080*	
C15B	0.55970 (10)	0.30295 (14)	0.87725 (10)	0.0594 (5)	
H15B	0.529711	0.350570	0.885427	0.071*	
C16A	0.31992 (12)	0.18082 (18)	0.51933 (11)	0.0812 (7)	
H16A	0.336601	0.186172	0.482145	0.097*	
C16B	0.57045 (12)	0.21752 (15)	0.91455 (10)	0.0701 (6)	
H16B	0.547553	0.207581	0.947049	0.084*	
C17A	0.25206 (11)	0.20883 (18)	0.51445 (11)	0.0788 (6)	
H17A	0.222830	0.232912	0.473896	0.095*	
C17B	0.61539 (12)	0.14692 (14)	0.90334 (10)	0.0670 (5)	
H17B	0.622904	0.089131	0.928349	0.080*	
C18A	0.22761 (10)	0.20122 (16)	0.56933 (10)	0.0664 (5)	
H18A	0.182157	0.221450	0.566455	0.080*	
C18B	0.64890 (11)	0.16181 (13)	0.85549 (9)	0.0597 (5)	
H18B	0.679967	0.114769	0.848603	0.072*	
C19A	0.27095 (9)	0.16322 (13)	0.62912 (9)	0.0503 (4)	
C19B	0.63656 (9)	0.24711 (12)	0.81718 (8)	0.0487 (4)	
C20A	0.27049 (8)	0.07604 (12)	0.72646 (8)	0.0460 (4)	
H20A	0.263258	0.016851	0.698369	0.055*	
C20B	0.67977 (8)	0.34979 (11)	0.74663 (8)	0.0455 (4)	
H20B	0.710566	0.381435	0.787027	0.055*	
C21A	0.35131 (8)	0.08860 (11)	0.75771 (8)	0.0428 (4)	
C21B	0.61020 (8)	0.41002 (11)	0.72630 (8)	0.0436 (4)	
C22A	0.38451 (8)	−0.00405 (12)	0.79834 (8)	0.0476 (4)	
H22A	0.350762	−0.057897	0.783413	0.057*	
C22B	0.62549 (9)	0.51980 (11)	0.71546 (8)	0.0455 (4)	
H22B	0.676045	0.530283	0.736231	0.055*	
C23A	0.40814 (11)	−0.00510 (16)	0.87480 (9)	0.0700 (6)	
H23A	0.368302	0.007301	0.891535	0.084*	
H23B	0.442939	0.045889	0.891870	0.084*	
C23B	0.60610 (12)	0.56658 (14)	0.64586 (9)	0.0653 (5)	
H23C	0.556070	0.558995	0.623743	0.078*	
H23D	0.631522	0.534571	0.618457	0.078*	
C24A	0.43983 (13)	−0.10576 (18)	0.89869 (11)	0.0858 (7)	
H24A	0.458127	−0.105855	0.947460	0.103*	
H24B	0.403309	−0.155412	0.885646	0.103*	
C24B	0.62518 (13)	0.67563 (15)	0.65357 (11)	0.0756 (6)	
H24C	0.675830	0.682527	0.671795	0.091*	
H24D	0.610696	0.706873	0.609649	0.091*	
C25A	0.49835 (11)	−0.13104 (16)	0.86895 (10)	0.0730 (6)	
H25A	0.537540	−0.086482	0.886693	0.088*	
H25B	0.514735	−0.197299	0.882073	0.088*	
C25B	0.58989 (11)	0.72645 (14)	0.69972 (10)	0.0636 (5)	
H25C	0.605044	0.794446	0.705938	0.076*	
H25D	0.539244	0.725563	0.679418	0.076*	
C26A	0.47364 (9)	−0.12383 (13)	0.79310 (9)	0.0567 (5)	
H26A	0.512746	−0.135699	0.775219	0.068*	
H26B	0.437793	−0.173014	0.774723	0.068*	
C26B	0.60860 (9)	0.67515 (12)	0.76724 (9)	0.0526 (4)	
H26C	0.658765	0.680734	0.789392	0.063*	
H26D	0.583857	0.705750	0.795785	0.063*	
C27A	0.22640 (9)	0.06439 (13)	0.77352 (8)	0.0487 (4)	
C28A	0.19594 (10)	0.14391 (15)	0.79585 (9)	0.0613 (5)	
H28A	0.202640	0.206892	0.781513	0.074*	
C29A	0.15615 (12)	0.13088 (18)	0.83872 (11)	0.0742 (6)	
H29A	0.135111	0.184939	0.852216	0.089*	
C30A	0.14685 (11)	0.0391 (2)	0.86210 (11)	0.0758 (6)	
C31A	0.17454 (11)	−0.04134 (18)	0.83929 (12)	0.0781 (6)	
H31A	0.167428	−0.104056	0.853796	0.094*	
C32A	0.21313 (10)	−0.02820 (15)	0.79449 (10)	0.0642 (5)	
H32A	0.230622	−0.082992	0.778022	0.077*	
N1A	0.44484 (6)	−0.02564 (9)	0.77373 (6)	0.0427 (3)	
N1B	0.58882 (7)	0.57169 (9)	0.75699 (6)	0.0422 (3)	
N2A	0.36328 (8)	0.18310 (11)	0.79905 (8)	0.0563 (4)	
N2B	0.56029 (8)	0.35936 (11)	0.66512 (8)	0.0554 (4)	
O1A	0.32294 (6)	−0.11223 (9)	0.66020 (6)	0.0587 (3)	
O1B	0.72347 (6)	0.50202 (9)	0.86615 (6)	0.0570 (3)	
O2A	0.24319 (6)	0.15690 (9)	0.68239 (6)	0.0563 (3)	
O2B	0.66760 (7)	0.25215 (8)	0.76614 (6)	0.0551 (3)	
O3A	0.38209 (11)	0.25581 (11)	0.77596 (9)	0.1030 (6)	
O3B	0.51849 (9)	0.30057 (12)	0.67531 (7)	0.0853 (5)	
O4A	0.34989 (9)	0.18385 (12)	0.85182 (8)	0.0898 (5)	
O4B	0.56698 (8)	0.37447 (11)	0.60975 (7)	0.0736 (4)	
O5A	0.10816 (11)	0.03538 (18)	0.90686 (10)	0.1248 (7)	
C27B	0.7205 (4)	0.3453 (5)	0.6970 (3)	0.0462 (18)	0.739 (5)
C28B	0.7051 (4)	0.2848 (5)	0.6416 (3)	0.0641 (18)	0.739 (5)
H28B	0.669868	0.237860	0.635718	0.077*	0.739 (5)
C32B	0.77906 (17)	0.4073 (3)	0.70849 (18)	0.0493 (9)	0.739 (5)
H32B	0.792727	0.445090	0.747497	0.059*	0.739 (5)
C31B	0.81621 (16)	0.4127 (3)	0.66306 (17)	0.0554 (8)	0.739 (5)
H31B	0.855771	0.452769	0.671879	0.066*	0.739 (5)
C29B	0.7415 (2)	0.2927 (3)	0.5943 (2)	0.0547 (10)	0.739 (5)
H29B	0.729465	0.253408	0.555934	0.066*	0.739 (5)
C30B	0.79578 (18)	0.3597 (3)	0.60473 (18)	0.0543 (10)	0.739 (5)
O5B	0.83334 (10)	0.3747 (2)	0.56040 (11)	0.0872 (11)	0.739 (5)
C33B	0.8100 (5)	0.3359 (5)	0.49718 (19)	0.120 (2)	0.739 (5)
H33D	0.842041	0.352981	0.472371	0.180*	0.739 (5)
H33E	0.807307	0.265729	0.500147	0.180*	0.739 (5)
H33F	0.764164	0.361772	0.474512	0.180*	0.739 (5)
C30C	0.7756 (7)	0.3199 (7)	0.5848 (5)	0.071 (4)	0.261 (5)
C31C	0.7930 (8)	0.3979 (8)	0.6284 (7)	0.093 (6)	0.261 (5)
H31C	0.825093	0.444831	0.623408	0.112*	0.261 (5)
C29C	0.7286 (6)	0.2499 (8)	0.5939 (5)	0.054 (3)	0.261 (5)
H29C	0.718689	0.193041	0.567970	0.064*	0.261 (5)
C27C	0.7154 (9)	0.3417 (11)	0.6924 (7)	0.044 (5)	0.261 (5)
C28C	0.6974 (7)	0.2692 (10)	0.6437 (6)	0.037 (3)	0.261 (5)
H28C	0.659949	0.228793	0.644641	0.044*	0.261 (5)
C32C	0.7625 (6)	0.4060 (9)	0.6799 (5)	0.064 (3)	0.261 (5)
H32C	0.775175	0.459998	0.708356	0.077*	0.261 (5)
C33C	0.8139 (10)	0.3749 (10)	0.4920 (6)	0.088 (4)	0.261 (5)
H33G	0.828998	0.346416	0.456279	0.133*	0.261 (5)
H33H	0.772775	0.414295	0.473055	0.133*	0.261 (5)
H33I	0.850886	0.415265	0.519569	0.133*	0.261 (5)
O5C	0.7985 (8)	0.3018 (5)	0.5303 (5)	0.174 (6)	0.261 (5)

### 6'-(4-Methoxyphenyl)-6a'-nitro-6a',6b',7',9',10',12a'-hexahydro-2H,6'H,8'H-spiro[acenaphthylene-1,12'-chromeno[3,4-a]indolizin]-2-one (II) Atomic displacement parameters (Å^2^)

**Table:**

	*U*^11^	*U*^22^	*U*^33^	*U*^12^	*U*^13^	*U*^23^
C33A	0.118 (2)	0.221 (4)	0.104 (2)	−0.017 (3)	0.055 (2)	0.049 (3)
C1A	0.0380 (8)	0.0415 (9)	0.0464 (9)	0.0001 (7)	0.0060 (7)	−0.0003 (7)
C1B	0.0433 (8)	0.0379 (8)	0.0448 (9)	0.0023 (6)	0.0103 (7)	−0.0032 (7)
C2A	0.0458 (9)	0.0473 (9)	0.0396 (9)	0.0007 (7)	0.0076 (7)	−0.0014 (7)
C2B	0.0452 (8)	0.0399 (8)	0.0399 (9)	0.0006 (7)	0.0101 (7)	−0.0022 (7)
C3A	0.0672 (11)	0.0629 (12)	0.0453 (10)	−0.0053 (9)	0.0097 (9)	−0.0095 (9)
C3B	0.0495 (9)	0.0629 (11)	0.0445 (10)	−0.0003 (8)	0.0056 (8)	−0.0021 (8)
C4A	0.0940 (16)	0.0789 (14)	0.0477 (11)	0.0054 (12)	0.0207 (11)	−0.0126 (10)
C4B	0.0667 (12)	0.0880 (15)	0.0366 (10)	−0.0059 (10)	0.0100 (9)	−0.0078 (9)
C5A	0.0842 (15)	0.0859 (15)	0.0502 (11)	0.0204 (12)	0.0331 (11)	0.0082 (11)
C5B	0.0680 (12)	0.0772 (13)	0.0454 (10)	−0.0058 (10)	0.0238 (9)	−0.0120 (9)
C6A	0.0567 (10)	0.0590 (11)	0.0486 (10)	0.0130 (8)	0.0214 (8)	0.0180 (9)
C6B	0.0540 (9)	0.0490 (10)	0.0435 (9)	−0.0033 (7)	0.0197 (8)	−0.0044 (8)
C7A	0.0522 (10)	0.0785 (14)	0.0725 (14)	0.0085 (10)	0.0299 (10)	0.0268 (12)
C7B	0.0579 (11)	0.0627 (11)	0.0583 (12)	0.0011 (9)	0.0289 (9)	−0.0082 (9)
C8A	0.0433 (9)	0.0676 (13)	0.0813 (15)	−0.0093 (9)	0.0158 (9)	0.0210 (11)
C8B	0.0427 (9)	0.0698 (12)	0.0624 (12)	0.0050 (8)	0.0178 (8)	−0.0078 (9)
C9A	0.0456 (9)	0.0499 (10)	0.0610 (11)	−0.0076 (7)	0.0079 (8)	0.0058 (8)
C9B	0.0454 (9)	0.0622 (11)	0.0474 (10)	0.0025 (8)	0.0105 (8)	−0.0064 (8)
C10A	0.0379 (8)	0.0406 (8)	0.0451 (9)	0.0025 (6)	0.0079 (7)	0.0069 (7)
C10B	0.0445 (8)	0.0411 (8)	0.0400 (9)	0.0020 (7)	0.0123 (7)	−0.0040 (7)
C11A	0.0425 (8)	0.0439 (9)	0.0407 (9)	0.0041 (7)	0.0099 (7)	0.0071 (7)
C11B	0.0459 (8)	0.0380 (8)	0.0381 (8)	−0.0016 (6)	0.0128 (7)	−0.0026 (7)
C12A	0.0369 (7)	0.0378 (8)	0.0405 (9)	−0.0011 (6)	0.0080 (6)	0.0001 (7)
C12B	0.0399 (8)	0.0420 (8)	0.0366 (8)	0.0022 (6)	0.0101 (6)	−0.0052 (7)
C13A	0.0408 (8)	0.0392 (8)	0.0421 (9)	0.0009 (6)	0.0099 (7)	0.0010 (7)
C13B	0.0434 (8)	0.0429 (9)	0.0397 (9)	−0.0014 (7)	0.0105 (7)	−0.0081 (7)
C14A	0.0482 (9)	0.0464 (9)	0.0461 (10)	0.0052 (7)	0.0150 (7)	0.0071 (7)
C14B	0.0526 (9)	0.0417 (9)	0.0421 (9)	−0.0084 (7)	0.0132 (7)	−0.0066 (7)
C15A	0.0598 (11)	0.0842 (14)	0.0623 (12)	0.0224 (10)	0.0281 (10)	0.0286 (11)
C15B	0.0708 (12)	0.0508 (11)	0.0636 (12)	−0.0091 (9)	0.0307 (10)	−0.0035 (9)
C16A	0.0805 (14)	0.1118 (18)	0.0593 (13)	0.0331 (13)	0.0329 (11)	0.0383 (12)
C16B	0.0945 (15)	0.0601 (13)	0.0634 (13)	−0.0204 (11)	0.0351 (11)	−0.0010 (10)
C17A	0.0696 (13)	0.1061 (17)	0.0602 (13)	0.0260 (12)	0.0184 (11)	0.0340 (12)
C17B	0.0973 (15)	0.0454 (11)	0.0553 (12)	−0.0147 (10)	0.0180 (11)	0.0025 (9)
C18A	0.0509 (10)	0.0855 (14)	0.0622 (12)	0.0183 (10)	0.0158 (9)	0.0207 (11)
C18B	0.0807 (13)	0.0430 (10)	0.0507 (11)	0.0003 (9)	0.0121 (10)	−0.0029 (8)
C19A	0.0485 (9)	0.0563 (10)	0.0470 (10)	0.0070 (8)	0.0155 (8)	0.0074 (8)
C19B	0.0606 (10)	0.0434 (9)	0.0395 (9)	−0.0034 (8)	0.0108 (8)	−0.0058 (7)
C20A	0.0470 (9)	0.0486 (9)	0.0419 (9)	0.0051 (7)	0.0126 (7)	−0.0018 (7)
C20B	0.0535 (9)	0.0400 (9)	0.0410 (9)	0.0032 (7)	0.0107 (7)	−0.0058 (7)
C21A	0.0481 (8)	0.0417 (9)	0.0372 (8)	0.0021 (7)	0.0106 (7)	−0.0046 (7)
C21B	0.0495 (9)	0.0421 (9)	0.0364 (8)	0.0019 (7)	0.0084 (7)	−0.0076 (7)
C22A	0.0485 (9)	0.0474 (9)	0.0461 (10)	0.0013 (7)	0.0129 (7)	0.0044 (7)
C22B	0.0518 (9)	0.0439 (9)	0.0413 (9)	0.0053 (7)	0.0145 (7)	−0.0019 (7)
C23A	0.0767 (13)	0.0877 (15)	0.0461 (11)	0.0142 (11)	0.0187 (10)	0.0157 (10)
C23B	0.0923 (14)	0.0616 (12)	0.0473 (11)	0.0162 (10)	0.0290 (10)	0.0067 (9)
C24A	0.0932 (16)	0.1017 (18)	0.0651 (14)	0.0218 (13)	0.0273 (12)	0.0403 (13)
C24B	0.1044 (17)	0.0631 (13)	0.0685 (14)	0.0133 (11)	0.0398 (13)	0.0208 (11)
C25A	0.0659 (12)	0.0767 (14)	0.0724 (14)	0.0132 (10)	0.0141 (10)	0.0339 (11)
C25B	0.0795 (13)	0.0463 (10)	0.0662 (13)	0.0087 (9)	0.0236 (10)	0.0090 (9)
C26A	0.0525 (10)	0.0478 (10)	0.0674 (12)	0.0068 (8)	0.0141 (9)	0.0137 (9)
C26B	0.0587 (10)	0.0411 (9)	0.0579 (11)	0.0040 (8)	0.0172 (8)	−0.0023 (8)
C27A	0.0495 (9)	0.0541 (10)	0.0441 (9)	0.0036 (8)	0.0165 (8)	−0.0023 (8)
C28A	0.0729 (12)	0.0595 (11)	0.0572 (11)	0.0113 (9)	0.0282 (10)	−0.0017 (9)
C29A	0.0859 (15)	0.0831 (16)	0.0632 (13)	0.0219 (12)	0.0371 (12)	−0.0039 (12)
C30A	0.0720 (13)	0.1079 (19)	0.0574 (13)	0.0161 (12)	0.0344 (11)	0.0134 (12)
C31A	0.0748 (14)	0.0784 (15)	0.0916 (17)	0.0060 (11)	0.0407 (13)	0.0218 (13)
C32A	0.0627 (11)	0.0590 (12)	0.0786 (14)	0.0051 (9)	0.0329 (10)	0.0009 (10)
N1A	0.0425 (7)	0.0423 (7)	0.0406 (7)	0.0028 (5)	0.0081 (6)	0.0054 (6)
N1B	0.0484 (7)	0.0395 (7)	0.0384 (7)	0.0049 (6)	0.0127 (6)	−0.0026 (6)
N2A	0.0664 (9)	0.0497 (9)	0.0507 (9)	0.0029 (7)	0.0141 (7)	−0.0073 (7)
N2B	0.0612 (9)	0.0555 (9)	0.0433 (9)	0.0045 (7)	0.0061 (7)	−0.0119 (7)
O1A	0.0418 (6)	0.0598 (8)	0.0754 (9)	−0.0114 (5)	0.0189 (6)	−0.0147 (6)
O1B	0.0413 (6)	0.0716 (8)	0.0559 (7)	0.0046 (6)	0.0113 (5)	−0.0160 (6)
O2A	0.0507 (6)	0.0699 (8)	0.0507 (7)	0.0178 (6)	0.0187 (6)	0.0121 (6)
O2B	0.0748 (8)	0.0418 (6)	0.0523 (7)	0.0087 (6)	0.0245 (6)	−0.0013 (5)
O3A	0.1712 (18)	0.0495 (9)	0.1053 (13)	−0.0144 (10)	0.0669 (13)	−0.0157 (9)
O3B	0.1002 (11)	0.0800 (10)	0.0658 (9)	−0.0357 (9)	0.0096 (8)	−0.0199 (8)
O4A	0.1308 (14)	0.0838 (11)	0.0629 (10)	−0.0117 (9)	0.0410 (9)	−0.0272 (8)
O4B	0.0853 (10)	0.0923 (10)	0.0396 (7)	0.0054 (8)	0.0127 (7)	−0.0163 (7)
O5A	0.1276 (15)	0.177 (2)	0.0977 (14)	0.0317 (14)	0.0767 (12)	0.0416 (14)
C27B	0.050 (3)	0.045 (4)	0.041 (3)	0.002 (2)	0.009 (2)	−0.002 (2)
C28B	0.055 (2)	0.065 (3)	0.071 (4)	−0.0138 (19)	0.016 (2)	−0.011 (2)
C32B	0.0444 (15)	0.0466 (16)	0.051 (2)	0.0017 (12)	0.0045 (15)	−0.0137 (17)
C31B	0.0461 (15)	0.0484 (17)	0.068 (2)	−0.0019 (12)	0.0111 (15)	−0.0030 (14)
C29B	0.0517 (19)	0.065 (3)	0.0460 (19)	−0.003 (2)	0.0115 (14)	−0.016 (2)
C30B	0.0475 (16)	0.066 (3)	0.049 (2)	−0.0010 (18)	0.0131 (16)	0.0008 (18)
O5B	0.0664 (13)	0.135 (3)	0.0689 (15)	−0.0143 (12)	0.0334 (11)	−0.0017 (14)
C33B	0.179 (5)	0.131 (5)	0.081 (3)	−0.036 (5)	0.085 (4)	−0.016 (3)
C30C	0.098 (11)	0.052 (7)	0.084 (8)	0.002 (6)	0.061 (8)	−0.008 (5)
C31C	0.130 (13)	0.062 (8)	0.121 (13)	−0.029 (7)	0.089 (11)	−0.021 (7)
C29C	0.074 (7)	0.050 (6)	0.040 (5)	0.013 (5)	0.022 (4)	−0.005 (5)
C27C	0.044 (9)	0.030 (9)	0.061 (11)	0.006 (6)	0.020 (8)	−0.015 (7)
C28C	0.041 (6)	0.054 (6)	0.020 (5)	0.015 (4)	0.014 (4)	−0.015 (4)
C32C	0.074 (7)	0.052 (5)	0.066 (7)	−0.009 (5)	0.021 (6)	−0.030 (6)
C33C	0.091 (8)	0.119 (11)	0.057 (7)	−0.012 (7)	0.023 (6)	0.006 (6)
O5C	0.365 (17)	0.056 (5)	0.200 (10)	0.013 (7)	0.238 (12)	−0.003 (6)

### 6'-(4-Methoxyphenyl)-6a'-nitro-6a',6b',7',9',10',12a'-hexahydro-2H,6'H,8'H-spiro[acenaphthylene-1,12'-chromeno[3,4-a]indolizin]-2-one (II) Geometric parameters (Å, º)

**Table:**

C33A—O5A	1.365 (4)	C20B—C27B	1.491 (4)
C33A—H33A	0.9600	C20B—C27C	1.5031 (10)
C33A—H33B	0.9600	C20B—C21B	1.555 (2)
C33A—H33C	0.9600	C20B—H20B	0.9800
C1A—O1A	1.2139 (18)	C21A—N2A	1.526 (2)
C1A—C2A	1.471 (2)	C21A—C22A	1.552 (2)
C1A—C12A	1.570 (2)	C21B—N2B	1.526 (2)
C1B—O1B	1.2126 (18)	C21B—C22B	1.554 (2)
C1B—C2B	1.476 (2)	C22A—N1A	1.467 (2)
C1B—C12B	1.580 (2)	C22A—C23A	1.518 (2)
C2A—C3A	1.372 (2)	C22A—H22A	0.9800
C2A—C11A	1.405 (2)	C22B—N1B	1.4663 (19)
C2B—C3B	1.371 (2)	C22B—C23B	1.521 (2)
C2B—C11B	1.405 (2)	C22B—H22B	0.9800
C3A—C4A	1.397 (3)	C23A—C24A	1.528 (3)
C3A—H3A	0.9300	C23A—H23A	0.9700
C3B—C4B	1.405 (2)	C23A—H23B	0.9700
C3B—H3B	0.9300	C23B—C24B	1.529 (3)
C4A—C5A	1.368 (3)	C23B—H23C	0.9700
C4A—H4A	0.9300	C23B—H23D	0.9700
C4B—C5B	1.369 (3)	C24A—C25A	1.511 (3)
C4B—H4B	0.9300	C24A—H24A	0.9700
C5A—C6A	1.415 (3)	C24A—H24B	0.9700
C5A—H5A	0.9300	C24B—C25B	1.514 (3)
C5B—C6B	1.409 (2)	C24B—H24C	0.9700
C5B—H5B	0.9300	C24B—H24D	0.9700
C6A—C11A	1.400 (2)	C25A—C26A	1.509 (3)
C6A—C7A	1.414 (3)	C25A—H25A	0.9700
C6B—C11B	1.403 (2)	C25A—H25B	0.9700
C6B—C7B	1.413 (2)	C25B—C26B	1.512 (2)
C7A—C8A	1.355 (3)	C25B—H25C	0.9700
C7A—H7A	0.9300	C25B—H25D	0.9700
C7B—C8B	1.363 (3)	C26A—N1A	1.463 (2)
C7B—H7B	0.9300	C26A—H26A	0.9700
C8A—C9A	1.416 (3)	C26A—H26B	0.9700
C8A—H8A	0.9300	C26B—N1B	1.461 (2)
C8B—C9B	1.412 (2)	C26B—H26C	0.9700
C8B—H8B	0.9300	C26B—H26D	0.9700
C9A—C10A	1.365 (2)	C27A—C32A	1.384 (3)
C9A—H9A	0.9300	C27A—C28A	1.386 (2)
C9B—C10B	1.363 (2)	C28A—C29A	1.368 (3)
C9B—H9B	0.9300	C28A—H28A	0.9300
C10A—C11A	1.400 (2)	C29A—C30A	1.372 (3)
C10A—C12A	1.517 (2)	C29A—H29A	0.9300
C10B—C11B	1.403 (2)	C30A—C31A	1.372 (3)
C10B—C12B	1.515 (2)	C30A—O5A	1.373 (2)
C12A—N1A	1.4617 (19)	C31A—C32A	1.383 (3)
C12A—C13A	1.555 (2)	C31A—H31A	0.9300
C12B—N1B	1.461 (2)	C32A—H32A	0.9300
C12B—C13B	1.556 (2)	N2A—O4A	1.2028 (19)
C13A—C14A	1.514 (2)	N2A—O3A	1.207 (2)
C13A—C21A	1.539 (2)	N2B—O4B	1.2146 (19)
C13A—H13A	0.9800	N2B—O3B	1.217 (2)
C13B—C14B	1.507 (2)	C27B—C28B	1.374 (4)
C13B—C21B	1.538 (2)	C27B—C32B	1.399 (5)
C13B—H13B	0.9800	C28B—C29B	1.387 (5)
C14A—C19A	1.383 (2)	C28B—H28B	0.9300
C14A—C15A	1.387 (2)	C32B—C31B	1.363 (5)
C14B—C19B	1.385 (2)	C32B—H32B	0.9300
C14B—C15B	1.390 (2)	C31B—C30B	1.364 (5)
C15A—C16A	1.375 (3)	C31B—H31B	0.9300
C15A—H15A	0.9300	C29B—C30B	1.380 (5)
C15B—C16B	1.378 (3)	C29B—H29B	0.9300
C15B—H15B	0.9300	C30B—O5B	1.363 (4)
C16A—C17A	1.377 (3)	O5B—C33B	1.3632 (10)
C16A—H16A	0.9300	C33B—H33D	0.9600
C16B—C17B	1.378 (3)	C33B—H33E	0.9600
C16B—H16B	0.9300	C33B—H33F	0.9600
C17A—C18A	1.370 (3)	C30C—O5C	1.3620 (10)
C17A—H17A	0.9300	C30C—C31C	1.3718 (10)
C17B—C18B	1.367 (3)	C30C—C29C	1.3868 (10)
C17B—H17B	0.9300	C31C—C32C	1.3820 (10)
C18A—C19A	1.385 (2)	C31C—H31C	0.9300
C18A—H18A	0.9300	C29C—C28C	1.3819 (10)
C18B—C19B	1.388 (2)	C29C—H29C	0.9300
C18B—H18B	0.9300	C27C—C32C	1.3631 (10)
C19A—O2A	1.3789 (19)	C27C—C28C	1.3814 (6)
C19B—O2B	1.378 (2)	C28C—H28C	0.9300
C20A—O2A	1.4316 (19)	C32C—H32C	0.9300
C20A—C27A	1.504 (2)	C33C—O5C	1.3660 (7)
C20A—C21A	1.555 (2)	C33C—H33G	0.9600
C20A—H20A	0.9800	C33C—H33H	0.9600
C20B—O2B	1.4308 (19)	C33C—H33I	0.9600
			
O5A—C33A—H33A	109.5	C22A—C21A—C20A	111.00 (13)
O5A—C33A—H33B	109.5	N2B—C21B—C13B	108.21 (13)
H33A—C33A—H33B	109.5	N2B—C21B—C22B	114.86 (13)
O5A—C33A—H33C	109.5	C13B—C21B—C22B	105.42 (12)
H33A—C33A—H33C	109.5	N2B—C21B—C20B	107.13 (12)
H33B—C33A—H33C	109.5	C13B—C21B—C20B	110.77 (13)
O1A—C1A—C2A	127.15 (15)	C22B—C21B—C20B	110.44 (13)
O1A—C1A—C12A	124.73 (14)	N1A—C22A—C23A	109.40 (14)
C2A—C1A—C12A	108.06 (12)	N1A—C22A—C21A	103.48 (12)
O1B—C1B—C2B	126.86 (14)	C23A—C22A—C21A	121.79 (15)
O1B—C1B—C12B	125.27 (14)	N1A—C22A—H22A	107.1
C2B—C1B—C12B	107.81 (12)	C23A—C22A—H22A	107.1
C3A—C2A—C11A	119.79 (16)	C21A—C22A—H22A	107.1
C3A—C2A—C1A	132.64 (16)	N1B—C22B—C23B	109.15 (13)
C11A—C2A—C1A	107.55 (13)	N1B—C22B—C21B	102.97 (12)
C3B—C2B—C11B	119.70 (15)	C23B—C22B—C21B	122.32 (14)
C3B—C2B—C1B	132.94 (15)	N1B—C22B—H22B	107.2
C11B—C2B—C1B	107.35 (13)	C23B—C22B—H22B	107.2
C2A—C3A—C4A	118.24 (18)	C21B—C22B—H22B	107.2
C2A—C3A—H3A	120.9	C22A—C23A—C24A	108.55 (17)
C4A—C3A—H3A	120.9	C22A—C23A—H23A	110.0
C2B—C3B—C4B	118.10 (16)	C24A—C23A—H23A	110.0
C2B—C3B—H3B	121.0	C22A—C23A—H23B	110.0
C4B—C3B—H3B	121.0	C24A—C23A—H23B	110.0
C5A—C4A—C3A	122.14 (19)	H23A—C23A—H23B	108.4
C5A—C4A—H4A	118.9	C22B—C23B—C24B	108.76 (16)
C3A—C4A—H4A	118.9	C22B—C23B—H23C	109.9
C5B—C4B—C3B	122.20 (17)	C24B—C23B—H23C	109.9
C5B—C4B—H4B	118.9	C22B—C23B—H23D	109.9
C3B—C4B—H4B	118.9	C24B—C23B—H23D	109.9
C4A—C5A—C6A	121.40 (18)	H23C—C23B—H23D	108.3
C4A—C5A—H5A	119.3	C25A—C24A—C23A	111.41 (17)
C6A—C5A—H5A	119.3	C25A—C24A—H24A	109.3
C4B—C5B—C6B	121.26 (17)	C23A—C24A—H24A	109.3
C4B—C5B—H5B	119.4	C25A—C24A—H24B	109.3
C6B—C5B—H5B	119.4	C23A—C24A—H24B	109.3
C11A—C6A—C7A	115.75 (17)	H24A—C24A—H24B	108.0
C11A—C6A—C5A	115.45 (17)	C25B—C24B—C23B	111.26 (17)
C7A—C6A—C5A	128.79 (18)	C25B—C24B—H24C	109.4
C11B—C6B—C5B	115.69 (15)	C23B—C24B—H24C	109.4
C11B—C6B—C7B	115.91 (15)	C25B—C24B—H24D	109.4
C5B—C6B—C7B	128.40 (16)	C23B—C24B—H24D	109.4
C8A—C7A—C6A	120.24 (17)	H24C—C24B—H24D	108.0
C8A—C7A—H7A	119.9	C26A—C25A—C24A	111.15 (17)
C6A—C7A—H7A	119.9	C26A—C25A—H25A	109.4
C8B—C7B—C6B	120.42 (16)	C24A—C25A—H25A	109.4
C8B—C7B—H7B	119.8	C26A—C25A—H25B	109.4
C6B—C7B—H7B	119.8	C24A—C25A—H25B	109.4
C7A—C8A—C9A	122.98 (17)	H25A—C25A—H25B	108.0
C7A—C8A—H8A	118.5	C26B—C25B—C24B	110.15 (15)
C9A—C8A—H8A	118.5	C26B—C25B—H25C	109.6
C7B—C8B—C9B	122.44 (16)	C24B—C25B—H25C	109.6
C7B—C8B—H8B	118.8	C26B—C25B—H25D	109.6
C9B—C8B—H8B	118.8	C24B—C25B—H25D	109.6
C10A—C9A—C8A	118.35 (17)	H25C—C25B—H25D	108.1
C10A—C9A—H9A	120.8	N1A—C26A—C25A	109.06 (16)
C8A—C9A—H9A	120.8	N1A—C26A—H26A	109.9
C10B—C9B—C8B	118.79 (16)	C25A—C26A—H26A	109.9
C10B—C9B—H9B	120.6	N1A—C26A—H26B	109.9
C8B—C9B—H9B	120.6	C25A—C26A—H26B	109.9
C9A—C10A—C11A	118.54 (15)	H26A—C26A—H26B	108.3
C9A—C10A—C12A	131.79 (15)	N1B—C26B—C25B	109.17 (14)
C11A—C10A—C12A	109.66 (13)	N1B—C26B—H26C	109.8
C9B—C10B—C11B	118.77 (14)	C25B—C26B—H26C	109.8
C9B—C10B—C12B	131.63 (14)	N1B—C26B—H26D	109.8
C11B—C10B—C12B	109.59 (13)	C25B—C26B—H26D	109.8
C10A—C11A—C6A	124.12 (15)	H26C—C26B—H26D	108.3
C10A—C11A—C2A	112.91 (14)	C32A—C27A—C28A	117.54 (16)
C6A—C11A—C2A	122.97 (16)	C32A—C27A—C20A	120.22 (15)
C6B—C11B—C10B	123.66 (14)	C28A—C27A—C20A	122.21 (16)
C6B—C11B—C2B	123.04 (14)	C29A—C28A—C27A	120.80 (19)
C10B—C11B—C2B	113.30 (13)	C29A—C28A—H28A	119.6
N1A—C12A—C10A	112.44 (12)	C27A—C28A—H28A	119.6
N1A—C12A—C13A	100.29 (11)	C28A—C29A—C30A	120.90 (19)
C10A—C12A—C13A	114.64 (12)	C28A—C29A—H29A	119.5
N1A—C12A—C1A	114.32 (12)	C30A—C29A—H29A	119.5
C10A—C12A—C1A	101.79 (12)	C31A—C30A—C29A	119.59 (19)
C13A—C12A—C1A	113.97 (12)	C31A—C30A—O5A	124.6 (2)
N1B—C12B—C10B	111.91 (12)	C29A—C30A—O5A	115.8 (2)
N1B—C12B—C13B	99.61 (11)	C30A—C31A—C32A	119.3 (2)
C10B—C12B—C13B	114.35 (12)	C30A—C31A—H31A	120.3
N1B—C12B—C1B	115.69 (12)	C32A—C31A—H31A	120.3
C10B—C12B—C1B	101.85 (12)	C31A—C32A—C27A	121.70 (19)
C13B—C12B—C1B	114.06 (12)	C31A—C32A—H32A	119.2
C14A—C13A—C21A	114.02 (12)	C27A—C32A—H32A	119.2
C14A—C13A—C12A	117.08 (13)	C12A—N1A—C26A	116.69 (13)
C21A—C13A—C12A	104.95 (12)	C12A—N1A—C22A	108.19 (11)
C14A—C13A—H13A	106.7	C26A—N1A—C22A	112.40 (13)
C21A—C13A—H13A	106.7	C26B—N1B—C12B	117.40 (12)
C12A—C13A—H13A	106.7	C26B—N1B—C22B	112.99 (13)
C14B—C13B—C21B	113.91 (13)	C12B—N1B—C22B	108.58 (12)
C14B—C13B—C12B	118.47 (13)	O4A—N2A—O3A	122.30 (16)
C21B—C13B—C12B	104.28 (12)	O4A—N2A—C21A	118.74 (16)
C14B—C13B—H13B	106.5	O3A—N2A—C21A	118.83 (15)
C21B—C13B—H13B	106.5	O4B—N2B—O3B	123.28 (16)
C12B—C13B—H13B	106.5	O4B—N2B—C21B	119.06 (16)
C19A—C14A—C15A	117.55 (15)	O3B—N2B—C21B	117.48 (15)
C19A—C14A—C13A	120.92 (14)	C19A—O2A—C20A	113.28 (12)
C15A—C14A—C13A	121.38 (15)	C19B—O2B—C20B	114.61 (12)
C19B—C14B—C15B	117.21 (16)	C33A—O5A—C30A	119.8 (2)
C19B—C14B—C13B	121.26 (14)	C28B—C27B—C32B	118.1 (4)
C15B—C14B—C13B	121.32 (15)	C28B—C27B—C20B	124.9 (4)
C16A—C15A—C14A	121.41 (17)	C32B—C27B—C20B	116.9 (4)
C16A—C15A—H15A	119.3	C27B—C28B—C29B	120.9 (5)
C14A—C15A—H15A	119.3	C27B—C28B—H28B	119.5
C16B—C15B—C14B	121.84 (18)	C29B—C28B—H28B	119.5
C16B—C15B—H15B	119.1	C31B—C32B—C27B	120.6 (3)
C14B—C15B—H15B	119.1	C31B—C32B—H32B	119.7
C15A—C16A—C17A	119.90 (19)	C27B—C32B—H32B	119.7
C15A—C16A—H16A	120.1	C32B—C31B—C30B	120.5 (3)
C17A—C16A—H16A	120.1	C32B—C31B—H31B	119.8
C17B—C16B—C15B	119.55 (19)	C30B—C31B—H31B	119.8
C17B—C16B—H16B	120.2	C30B—C29B—C28B	119.2 (4)
C15B—C16B—H16B	120.2	C30B—C29B—H29B	120.4
C18A—C17A—C16A	120.01 (18)	C28B—C29B—H29B	120.4
C18A—C17A—H17A	120.0	O5B—C30B—C31B	116.3 (3)
C16A—C17A—H17A	120.0	O5B—C30B—C29B	123.5 (3)
C18B—C17B—C16B	120.03 (18)	C31B—C30B—C29B	120.1 (3)
C18B—C17B—H17B	120.0	C30B—O5B—C33B	119.7 (4)
C16B—C17B—H17B	120.0	O5B—C33B—H33D	109.5
C17A—C18A—C19A	119.64 (17)	O5B—C33B—H33E	109.5
C17A—C18A—H18A	120.2	H33D—C33B—H33E	109.5
C19A—C18A—H18A	120.2	O5B—C33B—H33F	109.5
C17B—C18B—C19B	120.01 (18)	H33D—C33B—H33F	109.5
C17B—C18B—H18B	120.0	H33E—C33B—H33F	109.5
C19B—C18B—H18B	120.0	O5C—C30C—C31C	127.4 (8)
O2A—C19A—C14A	121.64 (14)	O5C—C30C—C29C	113.2 (8)
O2A—C19A—C18A	116.90 (15)	C31C—C30C—C29C	119.3 (7)
C14A—C19A—C18A	121.46 (16)	C30C—C31C—C32C	119.3 (9)
O2B—C19B—C14B	122.48 (15)	C30C—C31C—H31C	120.3
O2B—C19B—C18B	116.09 (15)	C32C—C31C—H31C	120.3
C14B—C19B—C18B	121.33 (17)	C28C—C29C—C30C	116.3 (10)
O2A—C20A—C27A	108.26 (12)	C28C—C29C—H29C	121.9
O2A—C20A—C21A	109.37 (13)	C30C—C29C—H29C	121.9
C27A—C20A—C21A	118.07 (13)	C32C—C27C—C28C	111.3 (7)
O2A—C20A—H20A	106.9	C32C—C27C—C20B	126.9 (7)
C27A—C20A—H20A	106.9	C28C—C27C—C20B	121.6 (6)
C21A—C20A—H20A	106.9	C27C—C28C—C29C	127.4 (10)
O2B—C20B—C27B	109.3 (3)	C27C—C28C—H28C	116.3
O2B—C20B—C27C	107.6 (6)	C29C—C28C—H28C	116.3
O2B—C20B—C21B	110.78 (13)	C27C—C32C—C31C	125.5 (8)
C27B—C20B—C21B	117.2 (4)	C27C—C32C—H32C	117.2
C27C—C20B—C21B	114.5 (8)	C31C—C32C—H32C	117.2
O2B—C20B—H20B	106.3	O5C—C33C—H33G	109.5
C27B—C20B—H20B	106.3	O5C—C33C—H33H	109.5
C21B—C20B—H20B	106.3	H33G—C33C—H33H	109.5
N2A—C21A—C13A	108.75 (13)	O5C—C33C—H33I	109.5
N2A—C21A—C22A	114.12 (13)	H33G—C33C—H33I	109.5
C13A—C21A—C22A	105.15 (12)	H33H—C33C—H33I	109.5
N2A—C21A—C20A	107.29 (12)	C30C—O5C—C33C	122.8 (10)
C13A—C21A—C20A	110.53 (12)		
			
O1A—C1A—C2A—C3A	1.8 (3)	O2A—C20A—C21A—C22A	−172.51 (12)
C12A—C1A—C2A—C3A	179.36 (18)	C27A—C20A—C21A—C22A	63.16 (18)
O1A—C1A—C2A—C11A	−176.79 (16)	C14B—C13B—C21B—N2B	89.18 (15)
C12A—C1A—C2A—C11A	0.74 (17)	C12B—C13B—C21B—N2B	−140.22 (12)
O1B—C1B—C2B—C3B	−4.9 (3)	C14B—C13B—C21B—C22B	−147.46 (13)
C12B—C1B—C2B—C3B	178.04 (17)	C12B—C13B—C21B—C22B	−16.86 (15)
O1B—C1B—C2B—C11B	173.74 (16)	C14B—C13B—C21B—C20B	−27.98 (17)
C12B—C1B—C2B—C11B	−3.31 (17)	C12B—C13B—C21B—C20B	102.62 (14)
C11A—C2A—C3A—C4A	−0.8 (3)	O2B—C20B—C21B—N2B	−62.61 (16)
C1A—C2A—C3A—C4A	−179.30 (18)	C27B—C20B—C21B—N2B	63.7 (3)
C11B—C2B—C3B—C4B	−1.4 (3)	C27C—C20B—C21B—N2B	59.2 (5)
C1B—C2B—C3B—C4B	177.13 (17)	O2B—C20B—C21B—C13B	55.21 (16)
C2A—C3A—C4A—C5A	0.1 (3)	C27B—C20B—C21B—C13B	−178.5 (3)
C2B—C3B—C4B—C5B	0.5 (3)	C27C—C20B—C21B—C13B	177.0 (5)
C3A—C4A—C5A—C6A	0.9 (3)	O2B—C20B—C21B—C22B	171.62 (12)
C3B—C4B—C5B—C6B	0.6 (3)	C27B—C20B—C21B—C22B	−62.0 (3)
C4A—C5A—C6A—C11A	−1.1 (3)	C27C—C20B—C21B—C22B	−66.6 (5)
C4A—C5A—C6A—C7A	177.58 (19)	N2A—C21A—C22A—N1A	−107.23 (15)
C4B—C5B—C6B—C11B	−0.7 (3)	C13A—C21A—C22A—N1A	11.86 (15)
C4B—C5B—C6B—C7B	179.02 (19)	C20A—C21A—C22A—N1A	131.41 (13)
C11A—C6A—C7A—C8A	0.1 (3)	N2A—C21A—C22A—C23A	16.2 (2)
C5A—C6A—C7A—C8A	−178.53 (19)	C13A—C21A—C22A—C23A	135.25 (16)
C11B—C6B—C7B—C8B	−0.7 (3)	C20A—C21A—C22A—C23A	−105.20 (18)
C5B—C6B—C7B—C8B	179.56 (19)	N2B—C21B—C22B—N1B	109.79 (14)
C6A—C7A—C8A—C9A	−0.7 (3)	C13B—C21B—C22B—N1B	−9.23 (15)
C6B—C7B—C8B—C9B	0.6 (3)	C20B—C21B—C22B—N1B	−128.93 (13)
C7A—C8A—C9A—C10A	0.5 (3)	N2B—C21B—C22B—C23B	−13.2 (2)
C7B—C8B—C9B—C10B	0.1 (3)	C13B—C21B—C22B—C23B	−132.19 (16)
C8A—C9A—C10A—C11A	0.4 (2)	C20B—C21B—C22B—C23B	108.11 (17)
C8A—C9A—C10A—C12A	179.46 (16)	N1A—C22A—C23A—C24A	−58.0 (2)
C8B—C9B—C10B—C11B	−0.7 (2)	C21A—C22A—C23A—C24A	−178.64 (16)
C8B—C9B—C10B—C12B	−179.43 (16)	N1B—C22B—C23B—C24B	57.3 (2)
C9A—C10A—C11A—C6A	−1.0 (2)	C21B—C22B—C23B—C24B	177.36 (16)
C12A—C10A—C11A—C6A	179.73 (14)	C22A—C23A—C24A—C25A	55.1 (3)
C9A—C10A—C11A—C2A	178.31 (14)	C22B—C23B—C24B—C25B	−56.0 (2)
C12A—C10A—C11A—C2A	−0.98 (18)	C23A—C24A—C25A—C26A	−54.5 (3)
C7A—C6A—C11A—C10A	0.7 (2)	C23B—C24B—C25B—C26B	55.9 (2)
C5A—C6A—C11A—C10A	179.58 (16)	C24A—C25A—C26A—N1A	55.5 (2)
C7A—C6A—C11A—C2A	−178.49 (15)	C24B—C25B—C26B—N1B	−56.4 (2)
C5A—C6A—C11A—C2A	0.3 (2)	O2A—C20A—C27A—C32A	144.97 (16)
C3A—C2A—C11A—C10A	−178.71 (15)	C21A—C20A—C27A—C32A	−90.2 (2)
C1A—C2A—C11A—C10A	0.12 (18)	O2A—C20A—C27A—C28A	−33.0 (2)
C3A—C2A—C11A—C6A	0.6 (2)	C21A—C20A—C27A—C28A	91.9 (2)
C1A—C2A—C11A—C6A	179.43 (14)	C32A—C27A—C28A—C29A	2.0 (3)
C5B—C6B—C11B—C10B	179.90 (16)	C20A—C27A—C28A—C29A	−179.94 (17)
C7B—C6B—C11B—C10B	0.1 (2)	C27A—C28A—C29A—C30A	1.7 (3)
C5B—C6B—C11B—C2B	−0.3 (2)	C28A—C29A—C30A—C31A	−3.7 (3)
C7B—C6B—C11B—C2B	179.99 (15)	C28A—C29A—C30A—O5A	177.6 (2)
C9B—C10B—C11B—C6B	0.6 (2)	C29A—C30A—C31A—C32A	1.8 (3)
C12B—C10B—C11B—C6B	179.57 (14)	O5A—C30A—C31A—C32A	−179.5 (2)
C9B—C10B—C11B—C2B	−179.31 (15)	C30A—C31A—C32A—C27A	2.0 (3)
C12B—C10B—C11B—C2B	−0.28 (19)	C28A—C27A—C32A—C31A	−3.8 (3)
C3B—C2B—C11B—C6B	1.3 (2)	C20A—C27A—C32A—C31A	178.07 (17)
C1B—C2B—C11B—C6B	−177.54 (14)	C10A—C12A—N1A—C26A	−66.24 (17)
C3B—C2B—C11B—C10B	−178.82 (15)	C13A—C12A—N1A—C26A	171.53 (13)
C1B—C2B—C11B—C10B	2.32 (18)	C1A—C12A—N1A—C26A	49.18 (17)
C9A—C10A—C12A—N1A	−55.1 (2)	C10A—C12A—N1A—C22A	165.86 (12)
C11A—C10A—C12A—N1A	124.09 (13)	C13A—C12A—N1A—C22A	43.63 (14)
C9A—C10A—C12A—C13A	58.6 (2)	C1A—C12A—N1A—C22A	−78.72 (15)
C11A—C10A—C12A—C13A	−122.21 (14)	C25A—C26A—N1A—C12A	173.59 (14)
C9A—C10A—C12A—C1A	−177.84 (16)	C25A—C26A—N1A—C22A	−60.58 (18)
C11A—C10A—C12A—C1A	1.32 (15)	C23A—C22A—N1A—C12A	−166.81 (14)
O1A—C1A—C12A—N1A	54.9 (2)	C21A—C22A—N1A—C12A	−35.61 (15)
C2A—C1A—C12A—N1A	−122.71 (13)	C23A—C22A—N1A—C26A	62.87 (18)
O1A—C1A—C12A—C10A	176.38 (15)	C21A—C22A—N1A—C26A	−165.92 (13)
C2A—C1A—C12A—C10A	−1.23 (15)	C25B—C26B—N1B—C12B	−171.60 (13)
O1A—C1A—C12A—C13A	−59.6 (2)	C25B—C26B—N1B—C22B	60.83 (18)
C2A—C1A—C12A—C13A	122.74 (14)	C10B—C12B—N1B—C26B	64.10 (17)
C9B—C10B—C12B—N1B	53.0 (2)	C13B—C12B—N1B—C26B	−174.65 (13)
C11B—C10B—C12B—N1B	−125.88 (14)	C1B—C12B—N1B—C26B	−51.95 (18)
C9B—C10B—C12B—C13B	−59.3 (2)	C10B—C12B—N1B—C22B	−166.23 (12)
C11B—C10B—C12B—C13B	121.81 (14)	C13B—C12B—N1B—C22B	−44.98 (14)
C9B—C10B—C12B—C1B	177.15 (17)	C1B—C12B—N1B—C22B	77.72 (15)
C11B—C10B—C12B—C1B	−1.70 (16)	C23B—C22B—N1B—C26B	−61.76 (18)
O1B—C1B—C12B—N1B	−52.5 (2)	C21B—C22B—N1B—C26B	166.88 (12)
C2B—C1B—C12B—N1B	124.62 (14)	C23B—C22B—N1B—C12B	166.17 (14)
O1B—C1B—C12B—C10B	−174.09 (15)	C21B—C22B—N1B—C12B	34.81 (15)
C2B—C1B—C12B—C10B	3.02 (15)	C13A—C21A—N2A—O4A	−167.26 (15)
O1B—C1B—C12B—C13B	62.2 (2)	C22A—C21A—N2A—O4A	−50.2 (2)
C2B—C1B—C12B—C13B	−120.69 (14)	C20A—C21A—N2A—O4A	73.17 (19)
N1A—C12A—C13A—C14A	−161.37 (13)	C13A—C21A—N2A—O3A	16.8 (2)
C10A—C12A—C13A—C14A	77.97 (17)	C22A—C21A—N2A—O3A	133.81 (18)
C1A—C12A—C13A—C14A	−38.77 (18)	C20A—C21A—N2A—O3A	−102.80 (19)
N1A—C12A—C13A—C21A	−33.80 (14)	C13B—C21B—N2B—O4B	159.85 (14)
C10A—C12A—C13A—C21A	−154.46 (12)	C22B—C21B—N2B—O4B	42.4 (2)
C1A—C12A—C13A—C21A	88.79 (15)	C20B—C21B—N2B—O4B	−80.67 (18)
N1B—C12B—C13B—C14B	164.26 (13)	C13B—C21B—N2B—O3B	−24.8 (2)
C10B—C12B—C13B—C14B	−76.27 (17)	C22B—C21B—N2B—O3B	−142.24 (16)
C1B—C12B—C13B—C14B	40.40 (19)	C20B—C21B—N2B—O3B	94.69 (17)
N1B—C12B—C13B—C21B	36.40 (14)	C14A—C19A—O2A—C20A	−32.1 (2)
C10B—C12B—C13B—C21B	155.88 (12)	C18A—C19A—O2A—C20A	148.56 (17)
C1B—C12B—C13B—C21B	−87.45 (15)	C27A—C20A—O2A—C19A	−168.62 (13)
C21A—C13A—C14A—C19A	5.9 (2)	C21A—C20A—O2A—C19A	61.49 (17)
C12A—C13A—C14A—C19A	128.88 (16)	C14B—C19B—O2B—C20B	28.4 (2)
C21A—C13A—C14A—C15A	−178.74 (16)	C18B—C19B—O2B—C20B	−155.07 (15)
C12A—C13A—C14A—C15A	−55.7 (2)	C27B—C20B—O2B—C19B	173.7 (3)
C21B—C13B—C14B—C19B	1.4 (2)	C27C—C20B—O2B—C19B	178.5 (8)
C12B—C13B—C14B—C19B	−121.74 (16)	C21B—C20B—O2B—C19B	−55.76 (17)
C21B—C13B—C14B—C15B	−173.16 (14)	C31A—C30A—O5A—C33A	19.1 (4)
C12B—C13B—C14B—C15B	63.7 (2)	C29A—C30A—O5A—C33A	−162.2 (3)
C19A—C14A—C15A—C16A	−1.9 (3)	O2B—C20B—C27B—C28B	47.4 (9)
C13A—C14A—C15A—C16A	−177.5 (2)	C21B—C20B—C27B—C28B	−79.6 (8)
C19B—C14B—C15B—C16B	0.1 (3)	O2B—C20B—C27B—C32B	−132.0 (5)
C13B—C14B—C15B—C16B	174.86 (17)	C21B—C20B—C27B—C32B	100.9 (6)
C14A—C15A—C16A—C17A	1.5 (4)	C32B—C27B—C28B—C29B	−7.3 (12)
C14B—C15B—C16B—C17B	0.7 (3)	C20B—C27B—C28B—C29B	173.3 (6)
C15A—C16A—C17A—C18A	0.2 (4)	C28B—C27B—C32B—C31B	5.1 (10)
C15B—C16B—C17B—C18B	0.0 (3)	C20B—C27B—C32B—C31B	−175.4 (4)
C16A—C17A—C18A—C19A	−1.4 (4)	C27B—C32B—C31B—C30B	1.8 (6)
C16B—C17B—C18B—C19B	−1.4 (3)	C27B—C28B—C29B—C30B	2.7 (10)
C15A—C14A—C19A—O2A	−178.68 (17)	C32B—C31B—C30B—O5B	175.8 (3)
C13A—C14A—C19A—O2A	−3.1 (3)	C32B—C31B—C30B—C29B	−6.5 (6)
C15A—C14A—C19A—C18A	0.7 (3)	C28B—C29B—C30B—O5B	−178.2 (5)
C13A—C14A—C19A—C18A	176.23 (17)	C28B—C29B—C30B—C31B	4.3 (7)
C17A—C18A—C19A—O2A	−179.63 (19)	C31B—C30B—O5B—C33B	−170.4 (5)
C17A—C18A—C19A—C14A	1.0 (3)	C29B—C30B—O5B—C33B	12.0 (7)
C15B—C14B—C19B—O2B	174.87 (15)	O5C—C30C—C31C—C32C	177.6 (13)
C13B—C14B—C19B—O2B	0.1 (2)	C29C—C30C—C31C—C32C	−1 (2)
C15B—C14B—C19B—C18B	−1.5 (2)	O5C—C30C—C29C—C28C	−173.1 (12)
C13B—C14B—C19B—C18B	−176.29 (15)	C31C—C30C—C29C—C28C	6 (2)
C17B—C18B—C19B—O2B	−174.41 (16)	O2B—C20B—C27C—C32C	−148.9 (17)
C17B—C18B—C19B—C14B	2.2 (3)	C21B—C20B—C27C—C32C	88 (2)
C14A—C13A—C21A—N2A	−94.40 (15)	O2B—C20B—C27C—C28C	38 (2)
C12A—C13A—C21A—N2A	136.20 (12)	C21B—C20B—C27C—C28C	−85.9 (18)
C14A—C13A—C21A—C22A	142.99 (13)	C32C—C27C—C28C—C29C	10 (3)
C12A—C13A—C21A—C22A	13.58 (15)	C20B—C27C—C28C—C29C	−175.7 (14)
C14A—C13A—C21A—C20A	23.14 (18)	C30C—C29C—C28C—C27C	−11 (2)
C12A—C13A—C21A—C20A	−106.27 (14)	C28C—C27C—C32C—C31C	−4 (3)
O2A—C20A—C21A—N2A	62.20 (16)	C20B—C27C—C32C—C31C	−178.3 (15)
C27A—C20A—C21A—N2A	−62.13 (18)	C30C—C31C—C32C—C27C	0 (3)
O2A—C20A—C21A—C13A	−56.24 (16)	C31C—C30C—O5C—C33C	−32 (3)
C27A—C20A—C21A—C13A	179.44 (13)	C29C—C30C—O5C—C33C	146.5 (16)

### 6'-(4-Methoxyphenyl)-6a'-nitro-6a',6b',7',9',10',12a'-hexahydro-2H,6'H,8'H-spiro[acenaphthylene-1,12'-chromeno[3,4-a]indolizin]-2-one (II) Hydrogen-bond geometry (Å, º)

*Cg*1, *Cg*2, *Cg*3 and *Cg*4 are the centroids of rings C27*A*–C32*A*, C14*B*–C19*B*, C27*B*–C32*B* and C2*B*–C6*B*/C11*B*, respecively.

**Table:**

*D*—H···*A*	*D*—H	H···*A*	*D*···*A*	*D*—H···*A*
C20*A*—H20*A*···O1*A*	0.98	2.38	3.225 (2)	144
C20*B*—H20*B*···O1*B*	0.98	2.28	3.152 (2)	147
C4*B*—H4*B*···O1*A*^i^	0.93	2.55	3.315 (2)	139
C25*A*—H25*A*···O5*B*^ii^	0.97	2.53	3.233 (3)	129
C9*B*—H9*B*···*Cg*1^iii^	0.93	2.87	3.709 (2)	150
C17*A*—H17*A*···*Cg*2^iv^	0.93	2.77	3.667 (2)	161
C26*B*—H26*C*···*Cg*3^v^	0.97	2.88	3.575 (3)	129
C33*B*—H33*F*···*Cg*4^ii^	0.96	2.86	3.707 (7)	148

Symmetry codes: (i) *x*+1/2, −*y*+1/2, *z*+1/2; (ii) −*x*+3/2, *y*−1/2, −*z*+3/2; (iii) −*x*+1/2, *y*+1/2, −*z*+3/2; (iv) *x*−3/2, −*y*−1/2, *z*−3/2; (v) −*x*+3/2, *y*+1/2, −*z*+3/2.
