# Supplementary material for: The crystal structures of 6′-(4-chloro­phen­yl)- and 6′-(4-meth­oxy­phen­yl)-6a′-nitro-6a′,6b′,7′,9′,10′,12a′-hexa­hydro-2H,6′H,8′H-spiro­[ace­naphthyl­ene-1,12′-chromeno[3,4-a]indolizin]-2-one
Source: Acta Crystallogr E Crystallogr Commun. 2019 Jan 15;75(Pt 2):218–22. doi: 10.1107/S2056989019000422 (PMC6362674; doi:10.1107/S2056989019000422)
Supplement: Supplementary file 4 [file e-75-00218-sup4.pdf]

Supplementary information file S1.

The puckering parameters (Cremer & Pople, 1975) and the lowest displacement asymmetry parameters (Nardelli, 1983) for compounds (I) and (II) – molecules A and B, are as follows:

For the five membered pyrrolidine ring q2 = 0.404 (2)Å, φ = 16.0 (1)°, ΔS(N1) is 13.4 (2)° and Δ2(C21) is 4.4 (2)° in (I), and q2 = 0.400 (2)Å, φ = 199.7 (2)°, ΔS(N1A) is 16.5 (2)° and Δ2(C21A) is 1.7 (2)° for molecule A, q2 = 0.414 (2)Å, φ = 23.9 (2)°, ΔS(C12B) is 14.0 (2)° and Δ2(C21B) is 5.5 (2)° for molecule B in (II).

For the six membered pyran ring q2 = 0.448 (2)Å, φ = 123.4 (3)°, ΔS(C14/C20) is 6.2 (2)° and Δ2(C19/C21) is 55.0 (2)° in (I), and,q2 = 0.435 (2)Å, φ =303.0 (2)°, ΔS(C14A/C20A) is 6.2 (2)° and Δ2(C19A/C21A) is 58.3 (2)° for molecule A, q2 = 0.377 (2)Å, φ =115.5 (2)°, ΔS(C14B/C20B) is 1.0 (2)° and Δ2(O2B/C13B) is 60.51 (2)° for molecule B in (II).

For the six membered piperidine ring q2 = 0.021 (2)Å, φ = 55 (2)°, ΔS(N1/C24) is 1.9 (2)° and Δ2 is 115.5 (2)° in (I), and q2 = 0.036 (2)Å, φ = 218 (2)°, ΔS(N1A/C24A) is 2.0 (2)° and Δ2 is 115.3 (2)° for molecule A, q2 = 0.019 (2)Å, φ = 38 (2)°, ΔS(N1B/C24B) is 0.7 (2)° and Δ2 is 116.0 (2)° for molecule B in (II).

Cremer, D. & Pople, J. A. (1975). *J. Am. Chem. Soc.* **97**, 1354--1358.

Nardelli, M. (1983). *Acta Cryst.* C**39**, 1141--1142.
